# Coping, escapism, and fantasy motives and depression symptoms mediate the relationship between emotion dysregulation and gaming disorder

**DOI:** 10.1016/j.abrep.2025.100663

**Published:** 2025-12-30

**Authors:** Christian Bäcklund, Daniel Eriksson Sörman, Hanna M. Gavelin, Orsolya Király, Zsolt Demetrovics, Jessica K. Ljungberg

**Affiliations:** a Department of Health, Education and Technology, Luleå University of Technology, Luleå, Sweden; b Department of Psychology, Umeå University, Umeå, Sweden; c Institute of Psychology, ELTE Eötvös Loránd University, Budapest, Hungary; d Centre of Excellence in Responsible Gaming, University of Gibraltar, Gibraltar; e Flinders University Institute for Mental Health and Wellbeing, College of Education, Psychology and Social Work, Flinders University, Bedford Park, SA, Australia

**Keywords:** Gaming disorder, Internet gaming disorder, Emotion dysregulation, Motivation, Depression

## Abstract

**Background:**

Studies have shown that emotion dysregulation, depression symptoms, and escapism motives are associated with Gaming Disorder (GD) symptoms. Findings indicate a discrepancy between the World Health Organization (WHO) and the American Psychiatric Association (APA) GD symptoms frameworks.

**Objective:**

The current study aimed to investigate the serial mediating effect of depression symptoms and coping, escapism and fantasy motives on the relationship between emotion dysregulation and GD symptoms and compare the WHO and APA frameworks.

**Methods:**

Data was collected through an online survey utilizing validated self-reported measures. The final convenience sample consisted of 678 video game players (68.44 % men *[n* = 464], 29.65 % women [*n* = 201], and 1.92 % reported ’other’ as gender identification [*n* = 13]) with an average age of 29.50 years (*SD* = 8.92). Structural equation modeling (SEM) was used to analyze the mediating effects.

**Results:**

The results showed that depression symptoms and a composite measure of coping, escapism, and fantasy motivations, in sequence, fully mediated the relationship between emotion dysregulation and GD symptoms within the WHO framework and partially mediated it within the APA framework.

**Conclusions:**

The results indicate that individuals with emotion dysregulation and higher levels of depression symptoms may use video games as an emotion regulation strategy. Managing emotion dysregulation and coping in the context of video games may aid in the clinical course for gaming disorder and co-occurring depression. Future research should utilize longitudinal designs to investigate study variables.

## Highlights

• The emotion dysregulation and GD symptoms relation was positive and significant.
• In the WHO model, depression and CEF motives fully mediated the relation.
• In the APA model, depression and CEF motives partially mediated the relation.

## 1 Introduction

The American Psychiatric Association (APA) acknowledged the potential issues of problematic gaming by including Internet Gaming Disorder (IGD) in Section III (“Emerging Measures and Models”) of the Diagnostic and Statistical Manual of Mental Disorders, Text Revision (DSM-5-TR; APA, 2022) as a condition requiring further investigation. In 2019, the World Health Organization (WHO) proposed an official diagnostic framework for problematic gaming with the inclusion of Gaming Disorder (GD) under the section of disorders due to addictive behaviors in the 11th revision of the International Classification of Diseases (ICD-11; WHO, 2019). The APA and WHO frameworks share common elements, such as criteria related to loss of control and significant impairments; they differ in the number of criteria and in the content of several criteria (see Table 1). Moreover, the WHO framework adopts a monothetic approach, requiring that all criteria be met for a diagnosis. In contrast, the APA uses a polythetic approach, where meeting five of the nine criteria is sufficient. This polythetic structure means that different symptom combinations can lead to the same diagnosis, resulting in considerable variability in how the disorder presents across individuals. Following the terminology of the ICD-11, the present study uses the term *gaming disorder symptoms* (GD symptoms) as an umbrella term when referring to symptoms of problematic gaming assessed across different diagnostic frameworks and measurement instruments in the literature.

**Table 1:** Comparison of APA and WHO Criteria for Gaming Disorder.

**Criteria**	**APA Framework (DSM-5-TR)**	**WHO Framework (ICD-11)**
Preoccupation with gaming	***✓***	**–**
Withdrawal symptoms when gaming is reduced or stopped	***✓***	**–**
Tolerance (needing to spend increasing amounts of time gaming)	***✓***	**–**
Impaired control over gaming	***✓***	***✓***
Increased priority of gaming and loss of interest in other activities	***✓***	***✓***
Continued gaming despite negative consequences	***✓***	***✓***
Deception regarding the amount of gaming	***✓***	**–**
Gaming to escape or relieve negative mood	***✓***	**–**
Significant impairments or negative consequences	***✓***	***✓***

*Note.***✓** = Included, **–** = Not included.

The inclusion of IGD and GD has generated a debate concerning the risks and benefits of establishing an official gaming-related diagnosis (e.g., Aarseth et al., 2017, Király et al., 2015, Király and Demetrovics, 2017; Van Den Brink, 2017; Van Rooij et al., 2018). Recent reviews indicate that problematic gaming behavior's global prevalence (i.e., potential risk group) may range from 1.96 % to 2.4 % (Kim et al., 2022; Stevens et al., 2020). However, earlier results have shown inconsistencies in estimating prevalence rates when comparing the frameworks of the WHO and the APA (Bäcklund et al., 2024a; Darvesh et al., 2020). Findings suggest that the WHO framework generates more conservative prevalence estimates in non-clinical samples (Bäcklund et al., 2024a, Ko et al., 2020, Montag et al., 2019, Pontes et al., 2022) and among individuals seeking treatment (Starcevic et al., 2020). A Delphi study involving international experts further indicates that some APA criteria may be less relevant for diagnostic purposes than those in the WHO framework (Castro-Calvo et al., 2021). Reviews and empirical work have also criticized several APA criteria, including mood modification (Griffiths et al., 2016, Ballou and Van Rooij, 2021) and tolerance (Razum et al., 2023). Taken together, these findings suggest that several APA criteria may risk over-pathologizing normal gaming. Considering that the frameworks proposed by the WHO and APA are widely used in research today (King et al., 2020, Wang and Cheng, 2020), additional studies comparing the APA and WHO frameworks are needed (Király, Potenza et al., 2022) to avoid possible biases in future studies on problematic gaming. The current study examines whether depression symptoms and gaming motivations mediate the association between emotion dysregulation and GD symptoms, using the APA and WHO frameworks tested in separate models.

### 1.1 Emotion regulation and gaming disorder symptoms

Emotion regulation refers to how individuals understand, accept, and influence their emotional states, whether through conscious or unconscious efforts (D’Agostino et al., 2017, Gratz et al., 2015, McRae and Gross, 2020). It is considered a transdiagnostic factor due to its relevance across a wide range of psychopathological conditions (Aldao et al., 2010, Daros et al., 2021; Stellern et al., 2023; Velotti et al., 2021; Visted et al., 2018), including behavioral addictions (González-Roz et al., 2024). Tull and Aldao (2015) distinguish between emotion regulation abilities, such as the capacity to manage emotions (e.g., maintaining control during stress), and emotion regulation strategies, such as the specific behaviors used to influence emotional experience (e.g., reappraisal or avoidance; Gross, 2015, McRae and Gross, 2020). These processes are connected, as individuals with reduced emotion regulation abilities tend to rely more on maladaptive strategies (Tull & Aldao, 2015). Research on GD has examined both abilities and strategies. Studies consistently show that greater difficulties concerning emotion regulation abilities are associated with higher GD symptoms (Estupiñá et al., 2024, Gisbert-Pérez et al., 2024, Marchica et al., 2019) and that individuals who lack adaptive strategies such as reappraisal appear more likely to meet criteria for GD (Yen et al., 2024). In the current study, difficulties with emotion regulation abilities reflect emotion dysregulation (Gratz & Roemer, 2004), and motivations for using video gaming to manage emotions were treated as a proxy for emotion regulation strategies, reflecting anticipated emotional effects of gaming (Demetrovics et al., 2011, Király et al., 2022a). In summary, the empirical evidence indicates that both emotion regulation abilities and strategies play a meaningful role in the development and maintenance of GD. However, other mental health symptoms may indirectly influence this association, such as depression, which may help clarify how emotion regulation difficulties relate to GD (Brand et al., 2016, Brand et al., 2019).

### 1.2 The mediating role of depression symptoms

Research from the mid-1990s suggested that difficulties with emotion regulation may contribute to the development of mental health issues (Gross, 1998, Gross and Muñoz, 1995). More recent work indicates an interplay between emotion dysregulation, depressive symptoms, and GD symptoms (Estupiñá et al., 2024, Marchica et al., 2020). The association between emotion dysregulation and depression is well established in systematic reviews (Hernández-Posadas et al., 2024, Sloan et al., 2017), a *meta*-analysis (Visted et al., 2018), and longitudinal studies (Berking et al., 2014; Masters et al., 2019). Specific emotion regulation strategies, such as reappraisal and acceptance, are generally associated with psychological well-being (Kraiss et al., 2020), while maladaptive strategies, such as emotional avoidance or suppression, are related to ill-being, including depression and addictive behaviors (González-Roz et al., 2024, Kraft et al., 2023, Lincoln et al., 2022). Evidence also indicates that rumination is a key maladaptive strategy associated with depression (Lincoln et al., 2022, Visted et al., 2018). Thus, individuals with difficulties in emotion regulation abilities may be more likely to rely on maladaptive strategies, including rumination, which may contribute to elevated depressive symptoms. Empirical evidence consistently shows a robust association between depressive symptoms and GD symptoms in cross-sectional (Gao et al., 2022, Ostinelli et al., 2021, Ropovik et al., 2023) and longitudinal studies (Amendola et al., 2025, Liu et al., 2018, Richard et al., 2020, Wang and Cheng, 2022). For some individuals, depressive symptoms may increase the appeal of gaming as a way to avoid or dull difficult emotional states. Taken together, individuals with emotion regulation difficulties and elevated depressive symptoms may be particularly likely to turn to gaming as a coping strategy, which may increase the risk of the development or maintenance of GD.

### 1.3 The mediating role of gaming motivations

Gaming motivation factors such as coping, escapism, and fantasy (CEF motives) reflect different urges to avoid difficult experiences, disengage from reality, or seek emotional relief (Demetrovics et al., 2011). Escapism captures the desire to step away from daily difficulties, coping reflects attempts to manage or reduce negative emotions, and fantasy involves the wish to enter an alternative world or adopt another identity. These motives reflect two common emotion regulation strategies: avoiding difficult emotional experiences and trying to suppress emotional discomfort (Lincoln et al., 2022, McRae and Gross, 2020). Theoretically, these motives reflect anticipated rather than experienced compensation during gaming (Brand et al., 2025). Empirical evidence shows that CEF motives are associated with GD symptoms (Bäcklund et al., 2022) and depressive symptoms (Laconi et al., 2017). In the present study, CEF motives are used as a composite indicator of gaming-related urges to regulate emotions. Taken together, these processes may be understood within broader theoretical models of addictive behavior that emphasize the interaction between individual vulnerabilities, affective processes, and reinforcement mechanisms.

### 1.4 The interaction Person affect cognition execution model

The current study draws on the Interaction Person Affect Cognition Execution (I-PACE) model to understand the relationship between the study variables (Brand et al., 2016, Brand et al., 2019, Brand et al., 2025). The I-PACE model is a comprehensive framework describing how addictive behaviors develop and are maintained. The model states that the addictive process involves an interaction between individual predisposing factors (e.g., temperamental features or psychopathological conditions such as depression) and affective responses to internal or external stimuli, including emotion regulation processes, which together influence decisions to engage in video game play. At different stages of GD development, gaming may involve varying degrees of positive reinforcement (gratification) and negative reinforcement (compensation), such as the excitement of in-game progress or the relief from difficult emotions. Over time, these reinforcement experiences may strengthen desire thinking and expectations that gaming is an effective way to obtain enjoyment or reduce distress (Brand et al., 2025). In short, individuals with greater emotional dysregulation, alone or combined with higher depressive symptoms, may be more vulnerable to negative emotional states and may rely on gaming for coping, which can contribute to GD over time.

### 1.5 Overview of the present study

**Fig. 1:**
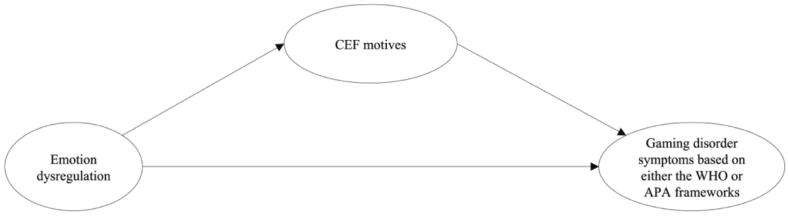
Visual overview of the proposed simple mediation models between emotion dysregulation and gaming disorder symptoms***.****Note.* GDT = The Gaming Disorder Test, IGDT-10 = Ten-Item Internet Gaming Disorder Test, CEF motives = Coping, Escapism, and Fantasy motives. Latent variables are depicted as circles. Individual measurement models of each latent factor can be seen in Appendix A.

Based on empirical evidence and the theoretical framework proposed by the I-PACE model, the current study investigated the mediating effect of CEF motives on the relationship between self-reported emotion dysregulation and self-assessed GD symptoms. Within the I-PACE framework, these motivations may reflect anticipated compensatory effects of gaming, whereby individuals expect gaming to reduce negative emotional states or provide emotional relief (Brand et al., 2025). In addition, the present study compared these associations across the WHO and APA frameworks. The first hypothesized model (prospectively registered: osf.io/Anonymous) of the study is shown in Fig. 1. It was hypothesized that CEF motives positively mediate the relationship between emotion dysregulation and GD symptoms (H1).

**Fig. 2:**
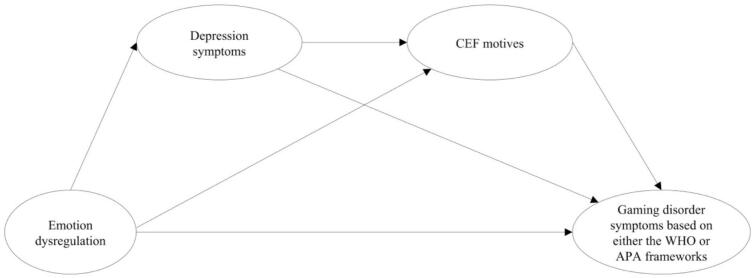
Visual overview of the proposed serial multiple mediation models between emotion dysregulation and gaming disorder symptoms**.***Note*. GDT = The Gaming Disorder Test, IGDT-10 = Ten-Item Internet Gaming Disorder Test, CEF motives = Coping, Escapism, and Fantasy motives. Circles represent latent variables. Separate measurement models of each latent factor can be seen in Appendix A.

The present study also investigated the mediating effects of depression symptoms and CEF motives in sequence on the association between emotion dysregulation and GD symptoms. This examination addressed a critical knowledge gap (Marchica et al., 2019), and the associations were also compared across the WHO and APA frameworks. The second hypothesized model, in which depressive symptoms were included, is illustrated in Fig. 2. It was hypothesized that depression symptoms (H2) and CEF motives (H3) may independently positively mediate the relationship between emotion dysregulation and GD symptoms in the same model and that depression symptoms and CEF motives may, in a sequence, positively mediate the relationship between emotion dysregulation and GD symptoms (H4).

## 2 Method

### 2.1 Participants and procedures

The present study was part of a research project at the Luleå University of Technology that used cross-sectional data and convenience sampling (osf.io/Anonymous; Anonymous et al., 2024). From September 13 to November 15, 2022, data was collected using an online survey that took approximately 15–30 min. The first 500 participants who completed the survey received a gift card valued at 50 SEK (approximately 4.5€). Incentives are often used to improve response rates (Singer & Couper, 2008), although recent work suggests that incentives have limited effects in online surveys (Wilson et al., 2024, Wu et al., 2022b). In the current study, participants who received the incentive did not differ significantly from those who did not concerning gender, age, years of education, or weekly gaming time, indicating that the incentive procedure did not introduce notable response bias. The current study utilized the following inclusion criteria: (I) fluent in the Swedish language, (II) aged at least 15 years old, and (III) engaging in video game play for a minimum of 10 h weekly on average. The 10-hour criterion was incorporated to focus on individuals with moderate to high gaming engagement, as those with infrequent play are generally at lower risk for problematic gaming behavior (Pontes et al., 2022). A total of 723 individuals completed the online survey, out of which 45 individuals were excluded from the study due to one of the following reasons: stated an average weekly gaming duration of less than 10 h (n = 35), did not report age (n = 3), reported ages below 15 years (n = 3), improbable values (n = 1) and did not complete most of the current study variables concerning gaming motivations (n = 3). Thus, the final sample consisted of 678 Swedish video game players. The current study was approved by the Swedish Ethical Review Authority (Dnr 2022-02393-01). Online informed consent was obtained from all participants after they were presented with an information letter describing the project and the nature of participation. All study procedures were conducted in accordance with the Declaration of Helsinki.

### 2.2 Measures

#### 2.2.1 Socio-demographics and gaming related characteristics

Data on socio-demographics and gaming-related characteristics included gender (68.44 percent men [n = 464], 29.65 percent women [*n* = 201], and 1.92 percent identifying as “other” [*n* = 13]), age (*M* = 29.50, *SD* = 8.92), and average weekly gaming time (*M* = 23.28, *SD* = 14.18), indicating a high gaming engagement but not necessarily pathological gaming. About one-third (32.27 %, *n* = 222) had completed a bachelor’s degree, and 51.9 % (*n* = 352) were working full time. Most preferred playing on a personal computer (70.01 %, *n* = 475). The most common primary game genre was MMORPGs (17.6 %, *n* = 124). For a complete description of the sociodemographic and gaming-related characteristics in the current sample, see Bäcklund et al. (2024).

#### 2.2.2 The gaming disorder test (GDT)

The Swedish version of the GDT was used to measure GD symptoms according to the WHO framework (Bäcklund et al., 2024a, Pontes et al., 2021). The GDT is a four-item self-report measure that operationalizes the three core criteria (e.g., “I have had difficulties controlling my gaming activity”), along with an additional item assessing significant life problems related to disordered gaming (WHO, 2019). The four items of the GDT are measured on a 5-point Likert scale (1 = never; 2 = rarely; 3 = sometimes; 4 = often; and 5 = very often). In the current study, GDT scores were analyzed as a continuous latent construct to capture variation in symptom severity. The total scores may range from 4 to 20, with higher scores indicating a higher degree of disordered gaming. A prior validation study conducted using the present sample supported the Swedish GDT's one-factor structure and reported prevalence estimates based on the recommended cutoff (Bäcklund et al., 2024a).

#### 2.2.3 Ten-item internet gaming disorder test (IGDT-10)

The Swedish version of the IGDT-10 was used to measure internet GD symptoms according to the APA framework (Bäcklund et al., 2024b, Király et al., 2017). The IGDT-10 is a ten-item self-report measure that operationalizes the nine diagnostic criteria for IGD proposed in the DSM-5-TR (e.g., “Have you ever unsuccessfully tried to reduce the time spent on gaming?”). The items are rated on a three-point scale (0 = never; 1 = sometimes; 2 = often). In the current study, responses of “often” were coded as 1 to indicate endorsement of a criterion, whereas responses of “never” and “sometimes” were coded as 0, consistent with the dichotomous scoring format of the DSM-5-TR. Responding “often” to either item 9, item 10, or both resulted in a score of one point, as these two items reflect the same criterion in the APA framework concerning negative consequences. In the current study, IGDT-10 scores were analyzed as a continuous latent construct to capture variation in symptom severity. The total scores may range from 0 to 9, with higher scores indicating a higher degree of GD symptoms. A prior validation study conducted using the present sample supported the Swedish IGDT-10’s one-factor structure and reported prevalence estimates based on the recommended cutoff (Bäcklund et al., 2024a).

#### 2.2.4 Difficulties in emotion regulation scale (DERS-16)

The DERS-16 (Bjureberg et al., 2016) is a brief 16-item self-report version of the original form of DERS (Gratz & Roemer, 2004), assessing emotion dysregulation across five domains, including lack of emotional clarity (e.g., “I have difficulty making sense out of my feelings”), difficulty engaging in goal-directed behaviors (e.g., “When I'm upset, I have difficulty focusing on other things”), impulse control difficulties (e.g., “When I'm upset, I feel out of control”), limited access to emotion regulation strategies (e.g., “When I'm upset, I believe that I will remain that way for a long time”), and nonacceptance of emotions (e.g., “When I'm upset, I feel ashamed with myself for feeling that way”). The questionnaire uses a 5-point Likert scale (1 = *almost never*; 2 = *sometimes*; 3 = *About half the time*; 4 = *Most of the time* and 5 = *almost always*) to represent the frequency of each statement. Thus, a total score on the entire scale may range from 16 to 80, with higher scores indicating more emotion dysregulation. The current study validated the factor structure through a second-order CFA (See Appendix A), which provided an excellent fit to the data after a minor modification.

#### 2.2.5 Patient health questionnaire (PHQ-9)

Depression symptoms over the last two weeks were measured with eight items (e.g., “Feeling down, depressed, or hopeless”) from the nine-item PHQ-9 (Kroenke et al., 2001). Item 9 from the original questionnaire covered thoughts of suicide and was therefore removed due to ethical considerations. Prior research indicates that omitting this item does not meaningfully affect the instrument's factor structure (Chae et al., 2025). A 4-point Likert scale was used to estimate the frequency of each symptom (0 = Not at all; 1 = Several days; 2 = More than half the days; 3 = Nearly every day). A total severity score may range from 0 to 24, where higher scores reflect a higher frequency of symptoms. The one-factor structure of the PHQ-9 was psychometrically investigated in the present study (See Appendix A).

#### 2.2.6 Motives for online gaming questionnaire (MOGQ)

The Swedish MOGQ (Bäcklund et al., 2024b, Demetrovics et al., 2011) is a self-reported questionnaire that assesses seven dimensions of gaming motivations (social, escapism, competition, coping, skill development, fantasy, and recreation). The present study included three of the gaming motivation factors: escapism (e.g., “because it makes me forget real life”), coping (e.g., “because it helps me relieve stress”) fantasy (e.g., “to feel as if I were someone else”) measured with four items each and answered on a 5-point Likert scale (1 = almost never/never; 2 = some of the time; 3 = half of the time; 4 = most of the time; and 5 = almost always/always). A total CEF motives score may range from 12 to 60, with higher scores indicating a higher frequency of these motivations. A second-order CFA was estimated to assess the appropriateness of utilizing three of the motivational factors from the MOGQ as a composite measure of CEF motives (see Appendix A).

### 2.3 Data management and statistical analysis

Descriptive statistics, including mean and standard deviation, were estimated based on the sum score of the included measures. Skewness and kurtosis were calculated to assess the normality of the study variables (Kim, 2013, Kline, 2015). Measurement models (i.e., confirmatory factor analyses) were estimated to assess the dimensionality of the included measures (see Appendix A). A full measurement model (see Fig. A4) was estimated where no structural relationships were specified, and all measures (including covariates: average weekly gaming time, age, and gender) were considered exogenous and correlated (i.e., a CFA that included all study measures). Women were coded as 0, and men were coded as 1 during the analyses. Interpretation of the standardized correlation coefficient was based on established guidelines (≥ 0.1 = small, ≥ 0.3 = moderate, and ≥ 0.5 = large) (Cohen, 1988). Structural equation modeling (SEM) was used to test the simple and serial multiple mediation models, examining the mediating effects of depression symptoms and CEF motives on the association between emotion dysregulation and GD symptoms. All SEM models, including the CFA, were conducted using the Weighted Least Square Mean and Variance Adjusted (WLSMV) estimator, suited for measures that include items with four or fewer response options (Rhemtulla et al., 2012) or when departures from multivariate normality are observed (Li, 2016a, Li, 2016b). The significance of indirect effects was tested by inspecting bootstrap 95 % confidence intervals (CI) (5000 bootstrap iterations), and were considered statistically significant if the CI did not include zero. Multiple goodness-of-fit indices were used to examine the model fit: a nonsignificant chi-square (χ^2^) value, Comparative Fit Index (CFI: near or above 0.95), Tucker-Lewis Fit Index (TLI: near or above 0.95), Standardized Root Mean Square Residual (SRMR: near or below 0.08), and Root Mean Square Error of Approximation (RMSEA: near or below 0.06 with a nonsignificant p-value [p ≤ 0.05]), including its 90 % confidence interval (90 % CI) and p-value (Hu and Bentler, 1999, Kline, 2015). All data management and descriptive statistics were conducted using IBM SPSS Statistics version 28 (IBM Corp., 2020). All SEM analyses were conducted using Mplus Version 8.8 (Muthén & Muthén, 1998–2017). There were 0–1.2 % missing responses across the items of the study variables (GDT, IGDT-10, PHQ-9, MOGQ, and DERS-16). Missing data in Mplus were handled using the default pairwise deletion (i.e., utilizing all available data), given the low percentage of missingness and its minimal expected impact (e.g., Madley-Dowd et al., 2019, Newman, 2014, Schafer, 1999).

#### 2.3.1 Sensitivity analyses

Several sensitivity analyses were conducted to assess the robustness of the findings in the present study (see Appendix B). The first series of sensitivity mediation analyses was conducted with the following covariates: average weekly gaming time, age, and gender. Only men and women were included as gender in the sensitivity analyses. Thus, the sensitivity analyses were conducted with part of the sample (*n* = 665) since missingness is not allowed for the observed covariates in Mplus (Muthén & Muthén, 2017). The second series of sensitivity analyses were only conducted on the models including the IGDT-10 but without item 8 (“Have you played to relieve a bad mood…”) considering the conceptual overlap with the escapism motivation factor.

## 3 Result

### 3.1 Descriptive statistics

Table 2 shows the descriptive statistics for the study measures. The distribution of GD symptoms (GDT and IGDT-10) showed positive skewness, suggesting that most participants report lower to moderate levels of gaming-related issues.

**Table 2:** Descriptive statistics of the included measures sum score (n = 678).

	Mean (SD)	Possible range	Skewness	Kurtosis
1. Gaming disorder symptoms				
GDT	7.92 (3.47)	4–20	0.92	0.42
IGDT-10	1.11 (1.71)	0–9	2.15	5.09
2. DERS_Total score_	32.22 (13.91)	16–80	0.95	0.31
Clarity	3.63 (1.96)	2–10	1.28	0.99
Goals	7.47 (3.39)	3–15	0.61	−0.60
Impulse	4.92 (2.72)	3–15	1.72	2.63
Strategies	9.80 (4.91)	5–25	1.05	0.38
Nonacceptance	6.41 (3.42)	3–15	0.88	−0.20
3. CEF motives_Total score_	26.08 (9.67)	12–60	0.68	−0.04
Escapism	8.92 (4.50)	4–20	0.82	−0.32
Coping	9.84 (3.33)	4–20	0.31	−0.24
Fantasy	7.31 (3.87)	4–20	1.30	1.08
4. Depression symptoms	6.16 (5.39)	0–24	0.97	0.39

*Note*. GDT = The Gaming Disorder Test (WHO); IGDT-10 = Ten-Item Internet Gaming Disorder Test (APA), DERS = Difficulties in Emotion Regulation Scale, CEF motives = Coping, Escapism, and Fantasy motives.

### 3.2 Simple mediation models between emotion dysregulation and gaming disorder symptoms

**Fig. 3:**
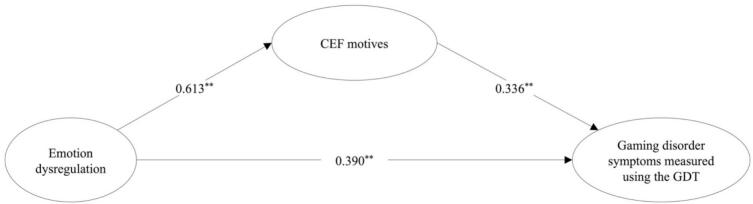
Simple mediation model between emotion dysregulation and gaming disorder symptoms (WHO)**.***Note*.^**^ Significant indicated by p < 0.001 and a bootstrap confidence interval that does not extend across zero. GDT = The Gaming Disorder Test. CEF motives = Coping, Escapism, and Fantasy motives. The GDT was used to assess gaming disorder symptoms according to the WHO framework.

**Fig. 4:**
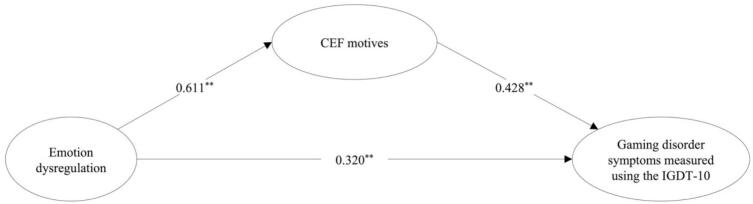
Simple mediation model between emotion dysregulation and gaming disorder symptoms (APA). *Note*.^**^ Significant indicated by p < 0.001 and a bootstrap confidence interval that does not extend across zero. IGDT-10 = Ten-Item Internet Gaming Disorder Test. CEF motives = Coping, Escapism, and Fantasy motives. The IGDT-10 was used to assess gaming disorder symptoms according to the APA framework.

The results showed that the two simple mediation models, Model_GDT_ and Model_IGDT-10_, fit the data well. The Model_GDT_, including the GDT as a measure of GD symptoms, showed the following fit indices: *χ^2^* = 1081.641, *df* = 453, *p* < 0.0001; CFI = 0.977; TLI = 0.974; RMSEA = 0.045 [0.042–0.049, *p* = 0.988], and SRMR = 0.044). The Model_IGDT-10_, including the IGDT-10 as a measure of GD symptoms showed the following fit statisics: *χ^2^* = 1240.978, df = 618, p < 0.0001; CFI = 0.977; TLI = 0.975; RMSEA = 0.039 [0.035–––0.042, *p* = 1.000], and SRMR = 0.060. Most standardized factor loadings for the latent variables in both models were large (*λ* > 0.60) and all were significant (*p* < 0.001). Table 3 shows the path coefficients from the two mediation models, Model_GDT_ and Model_IGDT-10_, including total, direct, and indirect effects (See Fig. 3, Fig. 4 for illustrations of the path coefficients). The positive direct effect of self-reported emotion dysregulation on self-assessed GD symptoms was statistically significant, as shown by the 95 % bootstrap confidence interval (Model_GDT_ = 0.282 to 0.491; Model_IGDT-10_ = 0.187 to 0.452). The mediating effect of CEF motives in the relationship between emotion dysregulation on GD symptoms was statistically significant in both models, as shown by the 95 % bootstrap confidence interval (Model_GDT_ = 0.138 to 0.283; Model_IGDT-10_ = 0.181 to 0.353). The results showed that the total model explained 42.6 % of the variance in GD symptoms in the Model_GDT_ and 45.2 % in the Model_IGDT-10_.

**Table 3:** Simple mediation models between emotion dysregulation and gaming disorder symptoms.

	Model_GDT_			Model_IGDT-10_		
**Path coefficients including direct effects**	Estimate *β* (S.E.)	*p* value	95 % CI	Estimate *β* (S.E.)	*p* value	95 % CI
Emotion dysregulation → Coping, Escapism, and Fantasy motives	0.613 (0.032)	<0.001	0.542–0.681	0.611 (0.032)	<0.001	0.541–0.680
Emotion dysregulation → Gaming disorder symptoms	0.390 (0.051)	<0.001	0.282–0.491	0.320 (0.064)	<0.001	0.187–0.452
Coping, Escapism, and Fantasy motives → Gaming disorder symptoms	0.336 (0.051)	<0.001	0.232–0.442	0.428 (0.058)	<0.001	0.308–0.553
**Indirect Effects**						
Emotion dysregulation → Coping, Escapism, and Fantasy motives → Gaming disorder symptoms	0.206 (0.034)	<0.001	0.138–0.283	0.262 (0.040)	<0.001	0.181–0.353
**Total effects**						
Emotion dysregulation → Gaming disorder symptoms	0.596 (0.031)	<0.001	0.529–0.658	0.581 (0.042)	<0.001	0.498–0.664

Note. Standardized coefficients (Estimate *β*) and the corresponding standard error (S.E.). GDT = The Gaming Disorder Test (WHO); IGDT-10 = Ten-Item Internet Gaming Disorder Test (APA).

### 3.3 Serial multiple mediation models between emotion dysregulation and gaming disorder symptoms

**Fig. 5:**
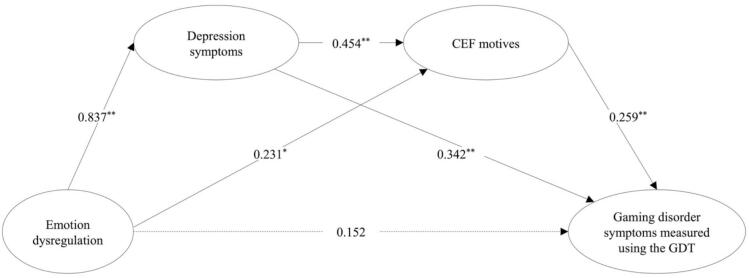
Serial multiple mediation model between emotion dysregulation and gaming disorder symptoms (WHO). *Note*. ^**^ Significant indicated by p < 0.001 and a bootstrap confidence interval that does not extend across zero. * Significant at level p < 0.05 and a bootstrap confidence interval that does not extend across zero. The dotted lines represent nonsignificant pathways. GDT = The Gaming Disorder Test. CEF motives = Coping, Escapism, and Fantasy motives. The GDT was used to assess gaming disorder symptoms according to the WHO framework.

**Fig. 6:**
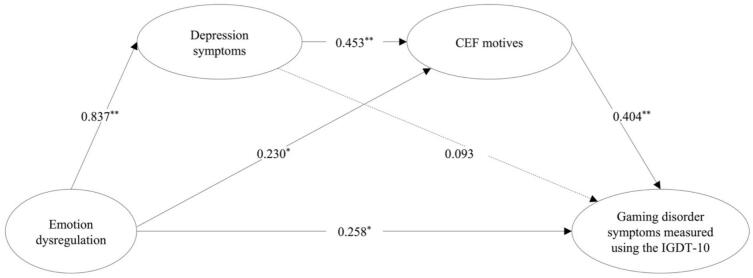
Serial multiple mediation model between emotion dysregulation and gaming disorder symptoms (APA). *Note*. ^**^ Significant indicated by p < 0.001 and a bootstrap confidence interval that does not extend across zero. * Significant at level p < 0.05 and a bootstrap confidence interval that does not extend across zero. The dotted lines represent nonsignificant pathways. IGDT-10 = Ten-Item Internet Gaming Disorder Test. CEF motives = Coping, Escapism, and Fantasy motives. The IGDT-10 was used to assess gaming disorder symptoms according to the APA framework.

The results showed that the two serial multiple mediation models, Model_SerialGDT_ and Model_SerialIGDT-10_, fit the data well. Model_SerialGDT_, including the GDT as a measure of GD symptoms, showed the following fit indices: *χ^2^* = 1803.014, *df* = 726, *p* < 0.0001; CFI = 0.966; TLI = 0.963; RMSEA = 0.047 [0.044–––0.049, *p* = 0.975], and SRMR = 0.050. Model_SerialIGDT-10_, including the IGDT-10 as a measure of GD symptoms with covariates showed the following fit statistics: *χ^2^* = 1897.816, df = 931, p < 0.0001; CFI = 0.970; TLI = 0.968; RMSEA = 0.039 [0.037–0.042, *p* = 1.000], and SRMR = 0.060. Table 4 shows the path coefficients from the two serial mediation models, Model_SerialGDT_ and Model_SerialIGDT-10_, including total, direct, and indirect effects (See Fig. 5, Fig. 6 for illustrations of the path coefficients). Most standardized factor loadings for the latent variables in both models were large (*λ* > 0.60) and all were significant (*p* < 0.001). The direct effect of self-reported emotion dysregulation on self-assessed GD symptoms was not significant in the Model_SerialGDT_ because the 95 % bootstrap confidence interval straddles zero (−0.042 to 0.324) and positively statistically significant in the Model_SerialIGDT10_, as shown by the 95 % bootstrap confidence interval (0.028 to 0.484). The indirect effect of emotion dysregulation on GD symptoms through only depression symptoms was significantly positive in the Model_SerialGDT_ as indicated by the bootstrap confidence interval above zero (0.135 to 0.451) and not significant in the Model_SerialIGDT-10_ because the bootstrap confidence interval extend across zero (−0.114 to 0.272). The indirect effect of emotion dysregulation on GD symptoms through only CEF motives was significantly positive in both models because the bootstrap confidence interval is above zero in both models (Model_SerialGDT_ = 0.013 to 0.121; Model_SerialIGDT-10_ = 0.021 to 0.184). The indirect effect of emotion dysregulation on GD symptoms through depression symptoms and CEF motives in a sequence was significantly positive in both models because the bootstrap confidence interval is above zero (Model_SerialGDT_ = 0.050 to 0.158; Model_SerialIGDT-10_ = 0.088 to 0.234). The total indirect effect, estimated as cumulative of the specific indirect effects statistically significant as indicated by the bootstrap confidence interval not containing zero (Model_SerialGDT_ = 0.300 to 0.612; Model_SerialIGDT-10_ = 0.133 to 0.523). The results showed that the total model explained 45.6 % of the variance in GD symptoms in the Model_GDT_ and 45.3 % in the Model_IGDT-10_.

**Table 4:** Serial multiple mediation models between emotion dysregulation and gaming disorder symptoms.

	Model_SerialGDT_			Model_SerialIGDT-10_		
**Path coefficients including direct effects**	Estimate *β* (S.E.)	*p* value	95 % CI	Estimate *β* (S.E.)	*p* value	95 % CI
Emotion dysregulation → Depression symptoms	0.837 (0.018)	<0.001	0.797–0.874	0.837 (0.018)	<0.001	0.797–0.874
Emotion dysregulation → Coping, Escapism, and Fantasy motives	0.231 (0.078)	0.003	0.057–0.400	0.230 (0.077)	0.003	0.057–0.398
Emotion dysregulation → Gaming disorder symptoms	0.152 (0.088)	0.085	−0.042–0.324	0.258 (0.110)	0.019	0.028–0.484
Depression symptoms → Coping, Escapism, and Fantasy motives	0.454 (0.077)	<0.001	0.286–0.620	0.453 (0.077)	<0.001	0.287–0.619
Depression symptoms → Gaming disorder symptoms	0.342 (0.090)	<0.001	0.162–0.532	0.093 (0.112)	0.407	−0.135–0.325
Coping, Escapism, and Fantasy motives → Gaming disorder symptoms	0.259 (0.052)	<0.001	0.149–0.370	0.404 (0.058)	<0.001	0.282–0.528
**Indirect Effects**						
Emotion dysregulation → Depression symptoms → Gaming disorder symptoms	0.286 (0.076)	<0.001	0.135–0.451	0.078 (0.094)	0.407	−0.114–0.272
Emotion dysregulation → Coping, Escapism, and Fantasy motives → Gaming disorder symptoms	0.060 (0.024)	0.015	0.013–0.121	0.093 (0.035)	0.008	0.021–0.184
Emotion dysregulation → Depression symptoms → Coping, Escapism, and Fantasy motives → Gaming disorder symptoms	0.098 (0.026)	<0.001	0.050–0.158	0.153 (0.034)	<0.001	0.088–0.234
**Total effects**						
Total effects	0.596 (0.031)	<0.001	0.528–0.658	0.582 (0.042)	<0.001	0.498–0.665
Total indirect	0.444 (0.074)	<0.001	0.300–0.612	0.324 (0.094)	0.001	0.133–0.523

*Note*. Standardized coefficients (Estimate *β*) and the corresponding standard error (S.E.). The Gaming Disorder Test (WHO), IGDT-10 = Ten-Item Internet Gaming Disorder Test (APA).

### 3.4 Sensitivity analyses

The results from the sensitivity analyses were mainly consistent with the primary analyses. When covariates were controlled for, the indirect effects of the mediation and serial multiple mediation analyses showed mostly consistent effect sizes and significance across the WHO and APA models. The serial mediation WHO model, including covariates, showed a marginal significant direct effect of emotion dysregulation on GD symptoms (*β* = 0.212, 95 % CI = 0.002 to 0.392), indicating partial mediation. In the mediation models with the IGDT-10 excluding Item 8, the indirect standardized effect sizes were consistent with the primary model.

## 4 Discussion

The present study aimed to investigate the mediating effect of depression symptoms and CEF motives on the relationship between self-reported emotion dysregulation and self-assessed GD symptoms across the WHO and APA GD symptoms frameworks. The results from the first hypothesized model (simple mediation) showed a significant direct association between emotion dysregulation and GD symptoms. The simple mediation models also demonstrated significant positive indirect effects of CEF motives between emotion dysregulation and GD symptoms across the WHO and APA frameworks, aligning with the present study’s first hypothesis (H1). These findings indicate that CEF motives partially mediate the relationship between emotion dysregulation and GD symptoms within the WHO and APA frameworks.

The results from the second hypothesized model (serial multiple mediations) showed that the direct relationship between emotion dysregulation and GD symptoms was not significant within the WHO framework and significant within the APA framework. In the WHO model, the results showed that depression symptoms (H2), CEF motives (H3), and depression symptoms and CEF motives in a sequence (H4) mediated the relationship. In the APA model, the results indicated that depression symptoms independently did not mediate the relationship, contrary to the second hypothesis (H2). However, CEF motives (H3) and depression symptoms and CEF motives in a sequence (H4) significantly mediated the relationship between emotion dysregulation and GD symptoms. These findings indicate that depression symptoms and CEF motives fully mediate the relationship between emotion dysregulation and GD symptoms within the WHO framework and partially mediate the association within the APA framework.

The results from the simple mediation models align with a prior study investigating the mediating role of escapism on the relationship between emotion dysregulation and GD symptoms among massively multiplayer online role-playing game players (Blasi et al., 2019). Based on the serial multiple mediation models, emotion dysregulation was positively associated with higher depression symptoms and CEF motives. Findings from cross-sectional and longitudinal studies have shown a relationship between emotion dysregulation and depression (Daros et al., 2021, Hernández-Posadas et al., 2024, Joormann and Stanton, 2016, Lincoln et al., 2022, Visted et al., 2018). The current study also indicated a positive relationship between emotion dysregulation, depression symptoms, CEF motives, and GD symptoms in a chained sequence. This result adds to similar studies showing that CEF motives mediate the relationship between psychiatric symptoms and GD symptoms (Ballabio et al., 2017, Bányai et al., 2019, Cudo et al., 2022, Király et al., 2015, Montag et al., 2019). Furthermore, *meta*-analytic results have shown a strong relationship between CEF motives and GD symptoms (Bäcklund et al., 2022). The findings indicate that individuals with difficulties in emotion regulation and higher depression symptoms play video games to avoid reality and cope with emotional states. The current study findings are consistent with research on substance use disorders, which has shown that coping motivations mediate the relationship between difficulties concerning emotion regulation abilities and alcohol use problems (Brown & Melas, 2024).

Research further suggests that gaming motivations may be better understood within a broader, multidimensional framework in which personal vulnerabilities, emotional processes, cognitive characteristics, and self-regulatory capacities interact. Within this perspective, problematic gaming has been associated with attachment-related difficulties and alexithymic traits, indicating that gaming may function as a maladaptive coping strategy for managing interpersonal distress and difficulties with emotional awareness and processing (Scalone et al., 2023). In this context, gaming may provide temporary relief from negative emotional states, which may, in turn, reinforce avoidance-oriented patterns of emotion regulation and contribute to their maintenance over time. Related findings from research on other behavioral addictions suggest that mechanisms such as compensation and escapism are not specific to gaming, but instead reflect more general processes through which individuals attempt to regulate psychological distress and disengage from aversive internal experiences, including within gaming-related motivational processes (Di Caro et al., 2025).

The present study contributes to the growing body of research on emotional processes that may underlie the development and maintenance of GD (Brand et al., 2025, Giardina et al., 2021). Drawing on the I-PACE model, individuals who experience difficulties in emotion regulation and symptoms of depression may be particularly susceptible to using video games as a means of managing negative affective states. At different stages of the addiction process, gaming may involve varying degrees of gratification and compensation, providing enjoyment while also relieving distress (Brand et al., 2025, Wegmann et al., 2025). These experiences may gradually strengthen desire thinking and the expectation that gaming is an effective way to regulate emotions, increasing the urge to continue playing even when negative consequences begin to emerge. With repeated pairing of gaming and mood relief, gaming may become a preferred and seemingly habitual coping strategy for dealing with aversive emotional states, which in some individuals may contribute to the development of GD (Brand et al., 2019, Brand et al., 2025). This emotional process aligns with the negative reinforcement model (Baker et al., 2004) and the self-medication hypothesis (Khantzian, 1997), which both suggest that addictive behaviors may develop as a way to mitigate negative emotions. The current study examined anticipated compensatory effects of gaming by using gaming motivations as a proxy for expected emotional regulation effects. The compensatory effect of gaming in specific situations may be more closely investigated using intensive longitudinal designs (Hamaker, 2025). Future research should examine day-to-day associations between cravings, compensation, gratification, and gaming patterns, similar to work on alcohol-related problems (Dora et al., 2023, Knapp et al., 2024, Wemm et al., 2019).

Research indicates that early life experiences may influence emotional dysregulation and vulnerability to problematic gaming (Kim et al., 2023), and emotion regulation and depression symptoms in a sequence mediate the relationship between childhood psychological maltreatment and GD symptoms (Wu, Liu et al., 2022). Furthermore, early life adversities and disrupted relationships from early childhood may negatively influence emotional regulation abilities, increasing reliance on external strategies to manage overwhelming feelings; within this framework, problematic gaming can be understood as a compensatory attempt to regulate distress or to distance oneself from trauma-related states (Santoro et al., 2025; Schimmenti et al., 2025). These findings may indicate that early-life adversities influenced the current study variables to varying degrees. Additionally, this interaction may take several forms, and since gamers are a heterogeneous population, the presence of co-occurring conditions may differ between individuals (Ko et al., 2023). Future research should utilize longitudinal research designs to provide knowledge regarding the role of emotion dysregulation in the onset and development of GD and related co-occurring psychiatric conditions, such as depression.

The current results showed that the mediating effects concerning depression symptoms and CEF motives were primarily consistent across the WHO and APA frameworks. This may be due to the similarities between the two diagnostic frameworks, such as overlapping criteria covering losing control over gaming, giving up other activities, and continuing despite adverse consequences. Furthermore, the analysis treated all included variables along a continuum (i.e., as composite latent variables). Thus, the associations with emotion dysregulation, depressive symptoms, and CEF motives may, primarily, be equally relevant across the entire spectrum of GD symptoms in both frameworks. The only dissimilarity between the serial multiple mediation models showed no significant direct effect between depression and (GD) symptoms in the APA model. This difference may be due to the unique criteria within the APA framework, such as preoccupation, withdrawal, and tolerance. Future studies may investigate the contributions of specific criteria within each diagnostic framework to external associations.

### 4.1 Practical implications

The current findings indicate that addressing emotion dysregulation and CEF motives in the context of video games may aid in the clinical course for GD and co-occurring depression. Additionally, learning specific emotion regulation techniques (e.g., mindfulness practices) may enhance life quality and minimize the risk of mental health issues reaching clinical severity (Blanchard et al., 2019, Menefee et al., 2022). Future research should investigate interventions to reduce emotional dependence on video games, potentially disrupting the addictive cycle (Dong et al., 2024).

### 4.2 Study limitations

First, the present study utilized an online convenience sample, which may not be representative of the general gaming population, individuals with higher levels of symptom severity, or clinical populations diagnosed with depression and/or GD. Additionally, the descriptive statistics indicate that most participants reported low to moderate GD symptom levels, which may have reduced observable variability and influenced the magnitude of the observed associations. Future studies should include representative random sampling and stratified random sampling for more accurate generalization of the findings. Additionally, future studies should investigate the interplay of emotion regulation abilities, depression, and the use of video games as an emotion regulation strategy within a clinical GD sample. Second, using self-reported measures opens to various response biases (e.g., memory recall and social desirability biases). Finally, the current study utilizes data from a cross-sectional survey where no causal inferences can be fully established. However, some argue that mediation models firmly grounded in theory may be applied to cross-sectional data without making any claims concerning the temporal ordering of the study measures (Hayes, 2022, Hayes and Rockwood, 2017). Future studies should utilize longitudinal and experimental research designs to establish covariation, temporal order, and removal of competing causes concerning the relationship between the study variables (i.e., examine evidence of causation), considering that emotional dysregulation may be a predictor and a consequence of psychopathology, including depression (Lincoln et al., 2022).

## 5 Conclusions

The current study showed that depression symptoms and CEF motives sequentially mediated the relationship between self-reported emotion dysregulation and self-assessed GD symptoms similarly across the WHO and APA diagnostic frameworks. The findings indicate that individuals with inadequate emotion regulation abilities and higher levels of depression symptoms may use video games as an emotion regulation strategy. Future research should utilize longitudinal and experimental designs to investigate study variables. Future intervention studies should investigate the effects of implementing adaptive emotion regulation strategies, which may address a core component of GD depression and improve psychological well-being.

## Funding sources

Jessica K. Ljungberg and Daniel Eriksson Sörman are supported by a grant from VINNOVA (grant number 2021–02361). Hanna Malmberg Gavelin is supported by the Swedish Research Council for Health, Working life and Welfare (grant number 2020–01111). Zsolt Demetrovics and Orsolya Király's contribution was supported by the Hungarian National Research, Development, and Innovation Office (KKP126835). None of these funding sources are related to this study, and the funding institutions/organizations had no role in the study design, data collection, analysis, interpretation, manuscript writing, or decision to submit the paper for publication.

## Data availability statement

The data that support the findings of this study are available on request from the corresponding author. The data are not publicly available due to privacy or ethical restrictions.

## Ethics approval statement

The current study was approved by the Swedish Ethical Review Authority (Dnr 2022-02393-01). An online informed consent was obtained from all participants after they had been informed about the project and what it means to participate. All study procedures were performed in accordance with the Declaration of Helsinki.

## CRediT authorship contribution statement

**Christian Bäcklund:** Writing – review & editing, Writing – original draft, Visualization, Project administration, Methodology, Formal analysis, Data curation, Conceptualization. **Daniel Eriksson Sörman:** Writing – review & editing, Methodology, Conceptualization. **Hanna M. Gavelin:** Writing – review & editing, Conceptualization. **Orsolya Király:** Writing – review & editing, Conceptualization. **Zsolt Demetrovics:** Writing – review & editing, Conceptualization. **Jessica K. Ljungberg:** Writing – review & editing, Conceptualization.

## Declaration of competing interest

The authors declare that they have no known competing financial interests or personal relationships that could have appeared to influence the work reported in this paper.

## Data Availability

The data that has been used is confidential.
