# Supplementary material for: Coping, escapism, and fantasy motives and depression symptoms mediate the relationship between emotion dysregulation and gaming disorder
Source: Addict Behav Rep. 2025 Dec 30;23:100663. doi: 10.1016/j.abrep.2025.100663 (PMC12813181; doi:10.1016/j.abrep.2025.100663)
Supplement: Supplementary Data 1 [file mmc1.docx]

**Appendix A. Descriptive item statistics and measurement models**

**Table A1.**

Descriptive Item statistics for the Difficulties in Emotion Regulation Scale (*n*=674 - 678)

|  | Mean | SD | Skewness | Kurtosis |
| --- | --- | --- | --- | --- |
| DERS 1 | 1.82 | 1.04 | 1.35 | 1.28 |
| DERS 2 | 1.82 | 1.09 | 1.28 | 0.74 |
| DERS 3 | 2.38 | 1.26 | 0.64 | -0.66 |
| DERS 4 | 1.59 | 0.97 | 1.86 | 3.06 |
| DERS 5 | 1.91 | 1.15 | 1.17 | 0.35 |
| DERS 6 | 1.75 | 1.12 | 1.42 | 0.98 |
| DERS 7 | 2.49 | 1.27 | 0.57 | -0.76 |
| DERS 8 | 1.68 | 1.09 | 1.62 | 1.67 |
| DERS 9 | 2.05 | 1.30 | 1.06 | -0.10 |
| DERS 10 | 2.07 | 1.29 | 1.00 | -0.22 |
| DERS 11 | 1.67 | 1.00 | 1.63 | 2.25 |
| DERS 12 | 1.91 | 1.91 | 1.21 | 0.42 |
| DERS 13 | 2.30 | 1.35 | 0.76 | -0.68 |
| DERS 14 | 2.03 | 1.30 | 1.13 | 0.07 |
| DERS 15 | 2.61 | 1.29 | 0.46 | -0.94 |
| DERS 16 | 2.22 | 1.31 | 0.80 | -0.58 |

*Note*. DERS = Difficulties in Emotion Regulation Scale.

*Confirmatory factor analys for the Difficulties in emotion regulation scale (DERS-16)*

The present study investigated the factor structure of the DERS-16 via a CFA since this was not included during the development of this scale (Bjureberg et al., 2016). The first second-order CFA provided an acceptable model fit (χ2 = 526.944, df = 99, p < .0001; CFI = 0.974; TLI = 0.969; RMSEA = 0.080 [0.073 - 0.087, p < 0.000], and SRMR = 0.036). However, the RMSEA value above the recommended threshold warranted inspection of residuals and modification indices. The residual values were all acceptable, but the modification indices indicated that Item 14 ("when I'm upset, I start to feel very bad about myself) showed high cross-loadings onto the nonacceptance factor (205.148). Considering the conceptual overlap between Item 14 and the three items within the nonacceptance factor (Items 9, 10, and 13), Item 14 was repositioned from the strategies factor to the nonacceptance factor for further investigation. Subsequently, the second second-order CFA model for DERS-16 showed an excellent fit to the data (χ2 = 359.159, df = 99, p < .0001; CFI = 0.984; TLI = 0.981; RMSEA = 0.062 [0.055 - 0.069, p = 0.002], and SRMR = 0.029). Inspection of modification indices in the modified model showed no indications of high cross-loadings. All standardized factor loadings were high and statistically significant (See Figure A1).

**Figure A1**

*Confirmatory factor analysis for the Difficulties in emotion regulation (n = 678)*


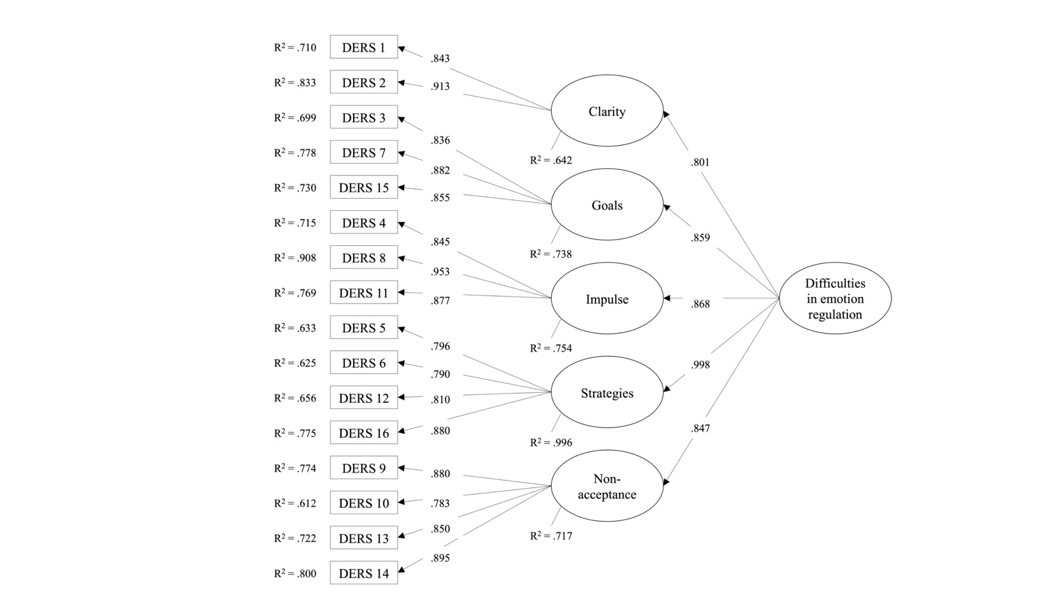


*Note*. DERS = Difficulties in Emotion Regulation Scale.

*Confirmatory factor analys for coping, escapism, and fantasy motives*

The second order CFA model containing the factors coping, escapism, and fantasy loadning onto an combined latent gaming motivation factor provided a good fit to the data: *χ^2^* = 214.472, df = 51, p < .0001; CFI = 0.986; TLI = 0.982; RMSEA = 0.069 [0.059 - 0.078, *p* = 0.001], and SRMR = 0.037. All standardized factor loadings were high and statistically significant (See Figure A2).

**Figure A2**

*Second-order confirmatory factor analysis for coping, escapism, and fantasy motives (n = 678)*


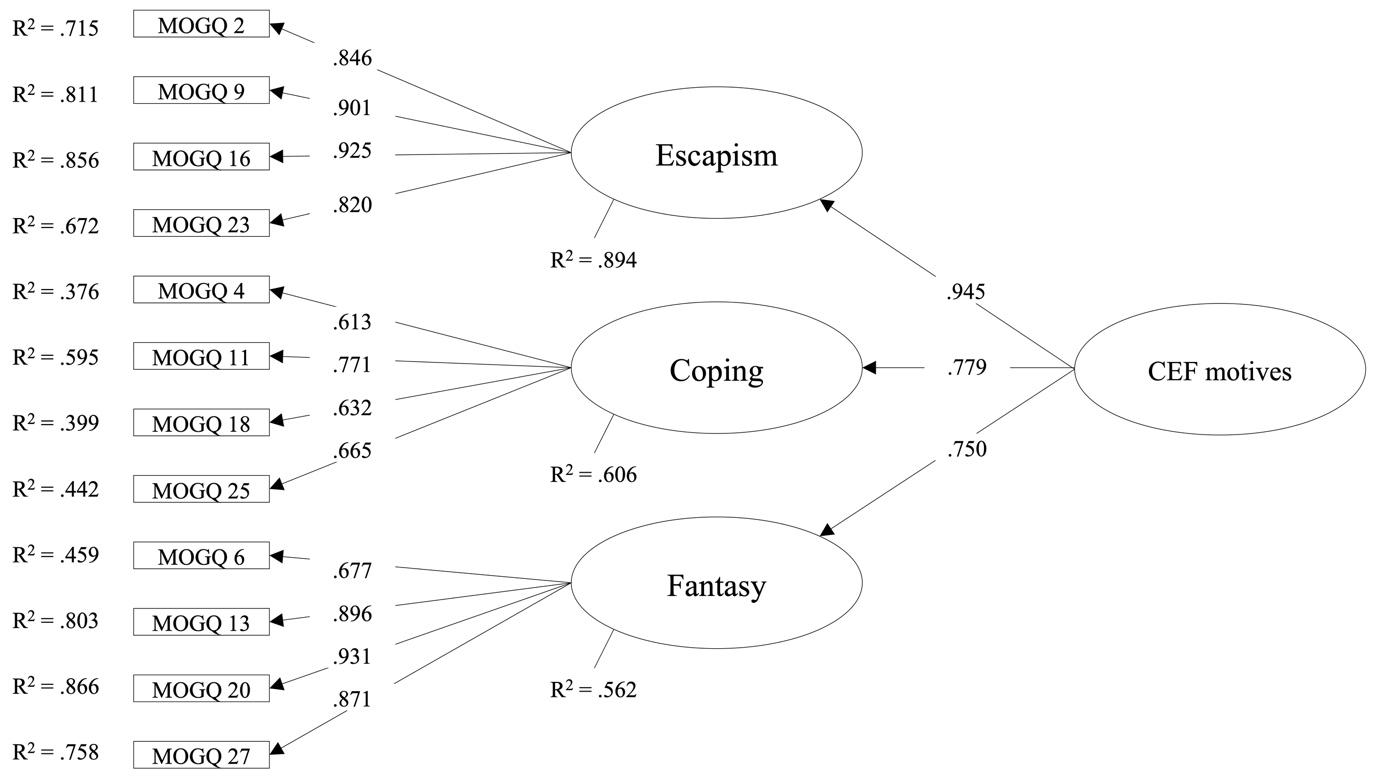


Note. MOGQ = Motives for online gaming questionnaire, CEF motives = Coping, Escapism, and Fantasy motives.

**Table A2.**

Descriptive Item statistics for the Patient Health Questionnaire (PHQ-9) (*n* = 672-677)

|  | Mean | SD | Skewness | Kurtosis |
| --- | --- | --- | --- | --- |
| PHQ1 | 0.73 | 0.90 | 1.13 | 0.41 |
| PHQ2 | 0.74 | 0.94 | 1.14 | 0.32 |
| PHQ3 | 1.05 | 1.05 | 0.64 | -0.81 |
| PHQ4 | 1.26 | 1.05 | 0.40 | -1.03 |
| PHQ5 | 0.83 | 1.06 | 1.00 | -0.37 |
| PHQ6 | 0.68 | 0.98 | 1.29 | 0.44 |
| PHQ7 | 0.61 | 0.92 | 1.41 | 0.93 |
| PHQ8 | 0.27 | 0.63 | 2.53 | 5.95 |

*Note*. PHQ = Patient Health Questionnaire

*Confirmatory factor analys for the Patient Health Questionnaire (PHQ-9)*

The CFA of the one-factor model for the PHQ-9 provided a good fit to the data: *χ^2^* = 160.429, df = 20, p < .0001; CFI = 0.969; TLI = 0.956; RMSEA = 0.102 [0.088 - 0.078, *p* < 0.001], and SRMR = 0.042. All standardized factor loadings were high and statistically significant (See Figure A3).

**Figure A3**

*Confirmatory factor analys for the Patient Health Questionnaire (PHQ-9) (n = 677)*

*
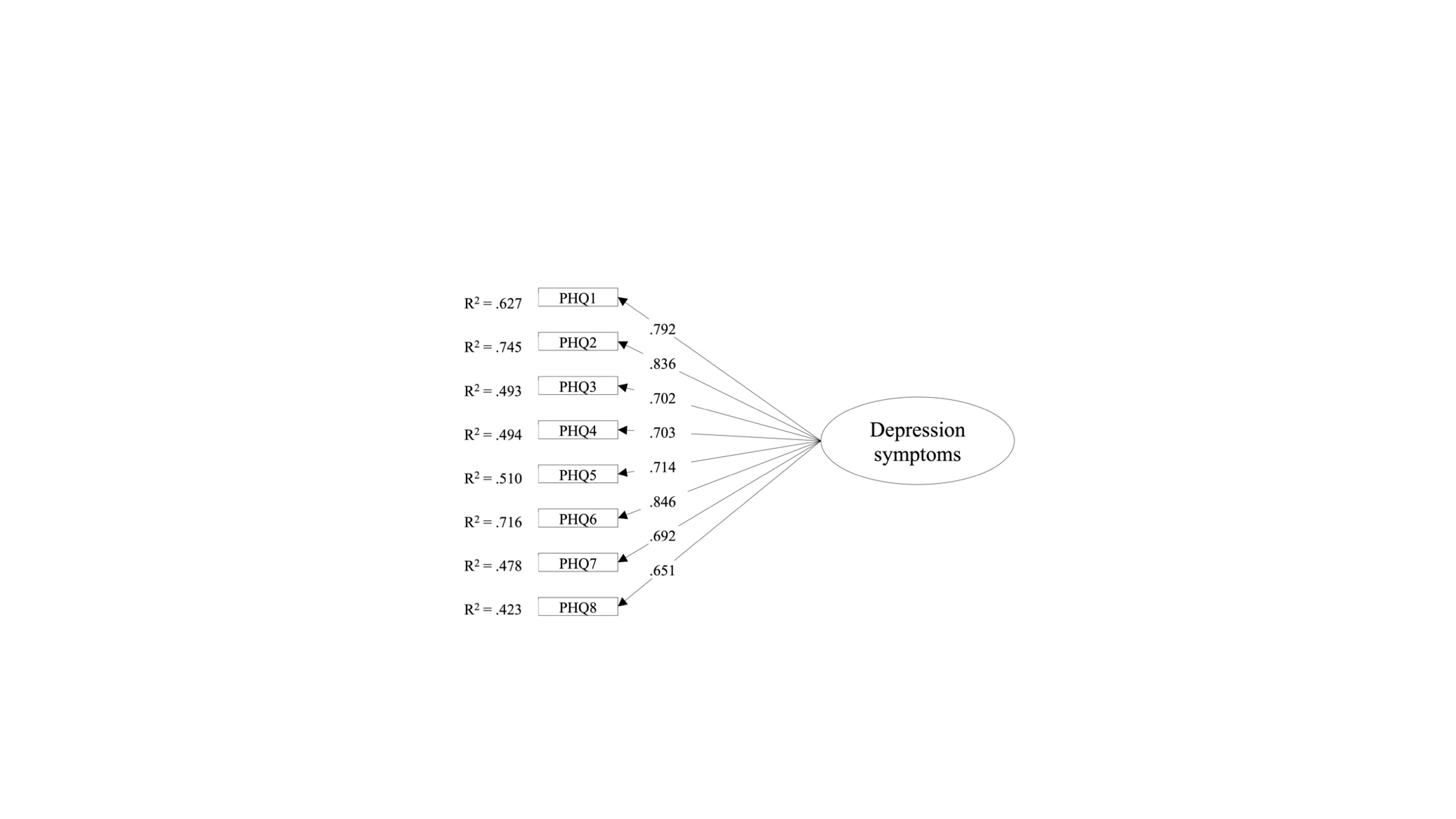
*

Note. PHQ = Patient Health Questionnaire

**Figure A4**

*Full measurement models (n = 678)*


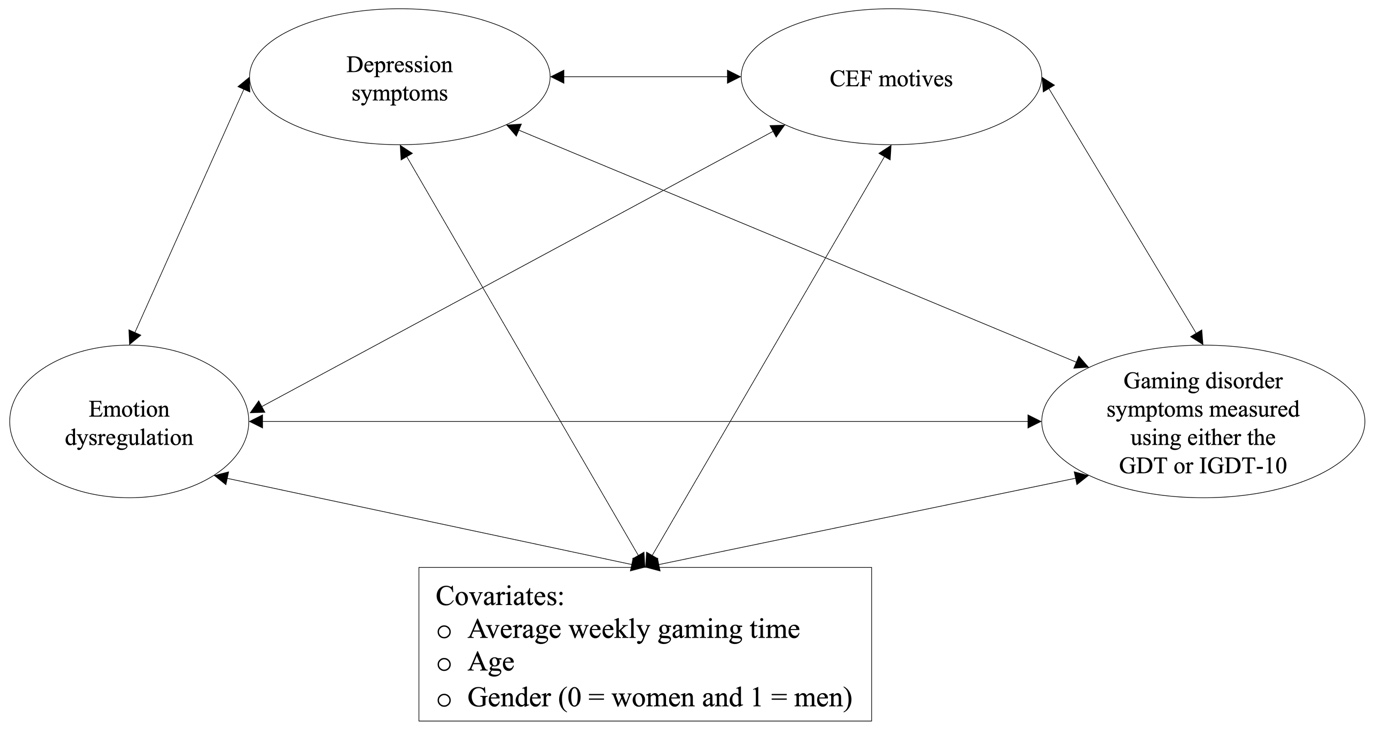


*Note*. GDT = The Gaming Disorder Test (WHO), IGDT-10 = Ten-Item Internet Gaming Disorder Test (APA), CEF motives = Coping, Escapism, and Fantasy motives.

**Full measurement models**

Figure 1 illustrates the full measurement models where no structural relationships were specified. The standardized correlation coefficients between the variables in the full measurement model are shown in Table 2. The full measurement models when gaming disorder symptoms were measured with the GDT (χ^2^ = 1962.680, df = 834, p < .0001; CFI = 0.965; TLI = 0.962; RMSEA = 0.048 [0.042 - 0.047, *p* = 1.000], and SRMR = 0.049) and with the IGDT-10 (χ^2^ = 2062.276, df = 1054, p < .0001; CFI = 0.969; TLI = 0.967; RMSEA = 0.038 [0.035 - 0.040, *p* = 1.000], and SRMR = 0.059) showed a good fit to the data. Gaming disorder symptoms measured with the GDT showed a large positive association with emotion dysregulation, depression symptoms, and CEF motives and a moderate positive relation with average weekly gaming time. Gaming disorder symptoms measured with the IGDT-10 also showed a large positive association with emotion dysregulation, depression symptoms, and CEF motives and a moderate positive relation with average weekly gaming time. Emotion dysregulation showed a large positive relationship with depression symptoms and CEF motives. Depression symptoms showed a large positive association with CEF motives. CEF motives showed a moderate positive association with average weekly gaming time.

**Table A3**

*Standardized correlations from the full measurement model*

|  | 1 | 2 | 3 | 4 | 5 | 6 |
| --- | --- | --- | --- | --- | --- | --- |
| 1. Gaming disorder symptoms (GDT / IGDT-10) | - |  |  |  |  |  |
| 2. Emotion dysregulation | .596^***^/ .582^***^ | - |  |  |  |  |
| 3. Depression symptoms | .636^***^/ .570^***^ | .837^***^ | - |  |  |  |
| 4. Coping, Escapism, and Fantasy motives | .573^***^/ .621^***^ | .611^***^ | .647^***^ | - |  |  |
| 5. Average weekly gaming time | .375^***^/ .352^***^ | .224^***^ | .278^***^ | .332^***^ | - |  |
| 6. Age | -.199^***^/ -.182^**^ | -.171^***^ | -.143^**^ | -.066 | -.092^***^ | - |
| 7. Gender^a^ | .185^**^/ .085 | -.187^**^ | -.098 | -.027 | .143^**^ | -.052 |

*Notes.* ^*^*p* < .05; ^**^ *p* < .01; ^***^ *p* < .001. GDT = The Gaming Disorder Test (WHO); IGDT-10 = Ten-Item Internet Gaming Disorder Test (APA). ^a^Coded as: 0 = Women, 1 = Men.

**Appendix B. Sensitivity analyses**

**Sensitivity analyses 1: Simple mediation models with covariates**

*Simple mediation models with covariates*

The results showed that the two mediation models, Model_GDTC_ and Model_IGDT-10C_, fit the data well. The Model_GDT_, including the GDT as a measure of gaming disorder symptoms and with covariates, showed the following fit indices: *χ^2^* = 1188.913, *df* = 540, *p* < 0.001; CFI = 0. 0.974; TLI = 0. 0.971; RMSEA = 0.043 [0.039– 0.046, *p* = 1.000]; and SRMR = 0.048. The Model_IGDT-10_, including the IGDT-10 as a measure of gaming disorder symptoms and with covaraites, showed the following fit statisics: *χ^2^* = 1370.983, *df* = 720, *p* < 0.001; CFI = 0.974; TLI = 0.972; RMSEA = 0.037 [0.034– 0.040, *p =* 1.000]; and SRMR = 0.061. Most standardized factor loadings for the latent variables in both models were large (*λ* > 0.60) and significant (*p* < 0.001). The results from the Model_GDTC_ showed that emotion dysregulation explained 41.8% of the variance of CEF motives and that the other variables in the model explained 50.4% of gaming disorder symptoms variance. The results from the Model_IGDT-10C_ showed that emotion dysregulation explained 41.1% of the variance of CEF motives and that the other variables in the model explained 50.9% of gaming disorder symptoms variance.

**Figure B1**

Visual overview of the paths included in the mediation model with covariates.


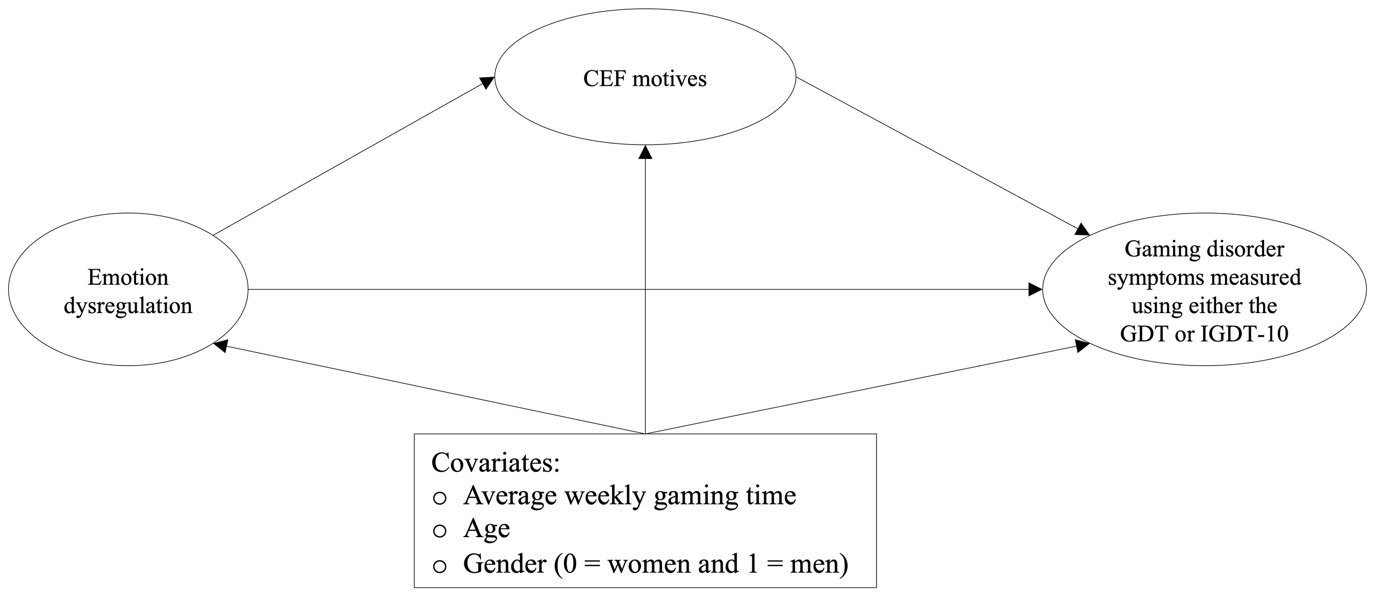


*Note*. GDT = The Gaming Disorder Test (WHO), IGDT-10 = Ten-Item Internet Gaming Disorder Test (APA), CEF motives = Coping, Escapism, and Fantasy motives. Individual measurement models of each latent factor can be seen in Appendix A.

**Table B1**

*Mediation model including covariates (5000 bootstrap) (N = 665).*

|  | Model_GDTC_ | | | Model_IGDT-10C_ | | |
| --- | --- | --- | --- | --- | --- | --- |
| **Path coefficients** | Estimate *β* (S.E.) | *p* value | 95% CI | Estimate *β* (S.E.) | *p*  value | 95% CI |
| Emotion dysregulation 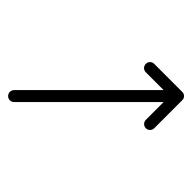 Coping, Escapism, and Fantasy motives | 0.576 (0.035) | < .001 | 0.497-0.651 | 0.574 (0.035) | < .001 | 0.495-0.650 |
| Emotion dysregulation 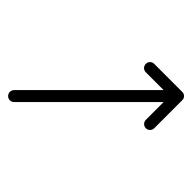 Gaming disorder symptoms | 0.416 (0.049) | < .001 | 0.300-0.525 | 0.341 (0.064) | < .001 | 0.196-0.482 |
| Coping, Escapism, and Fantasy motives 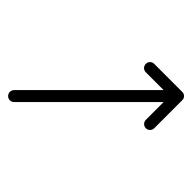 Gaming disorder symptoms | 0.260 (0.050) | < .001 | 0.156-0.370 | 0.364 (0.061) | < .001 | 0.232-0.503 |
| **Indirect Effects** |  |  |  |  |  |  |
| Emotion dysregulation 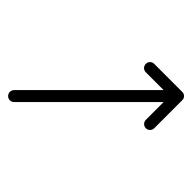 Coping, Escapism, and Fantasy motives 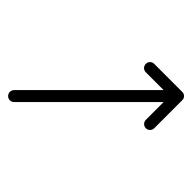 Gaming disorder symptoms | 0.150 (0.031) | < .001 | 0.089-0.223 | 0.209 (0.039) | < .001 | 0.130-0.303 |
| **Total effects** |  |  |  |  |  |  |
| Emotion dysregulation 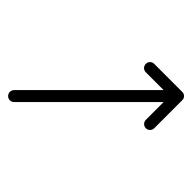 Gaming disorder symptoms | 0.566 (0.032) | < .001 | 0.488-0.638 | 0.549 (0.044) | < .001 | 0.458-0.644 |
| **Covariates** |  |  |  |  |  |  |
| Average weekly gaming time 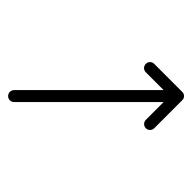 Gaming disorder symptoms | 0.168 (0.035) | < .001 | 0.085-0.252 | 0.158 (0.044) | < .001 | 0.061-0.246 |
| Average weekly gaming time 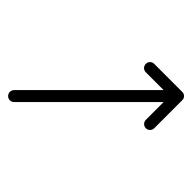 Emotion dysregulation | 0.237 (0.035) | < .001 | 0.158-0.310 | 0.237 (0.035) | < .001 | 0.158-0.310 |
| Average weekly gaming time 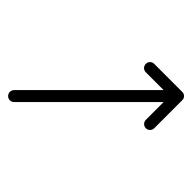 Coping, Escapism, and Fantasy motives | 0.203 (0.032) | < .001 | 0.132-0.275 | 0.202 (0.032) | < .001 | 0.132-0.275 |
| Age 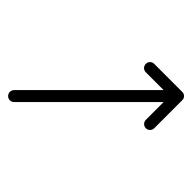 Gaming disorder symptoms | -0.081 (0.036) | .027 | -0.156-(-0.007) | -0.078 (0.051) | .124 | -0.185-0.009 |
| Age 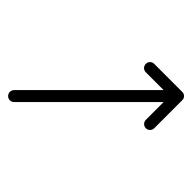 Emotion dysregulation | -0.153 (0.040) | < .001 | -0.234-(0.075) | -0.153 (0.040) | < .001 | -0.234-(-0.076) |
| Age 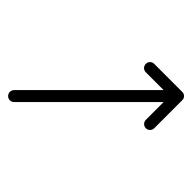 Coping, Escapism, and Fantasy motives | 0.061 (0.037) | .099 | -0.011-0.136 | 0.061 (0.037) | .099 | -0.011-0.136 |
| Gender 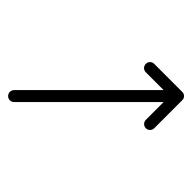 Gaming disorder symptoms | 0.184 (0.033) | < .001 | 0.119-0.250 | 0.087 (0.041) | .034 | 0.011-0.171 |
| Gender 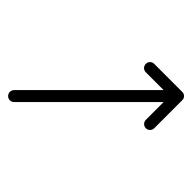 Emotion dysregulation | -0.172 (0.040) | < .001 | -0.248-(-0.089) | -0.172 (0.040) | < .001 | -0.248-(-0.089) |
| Gender 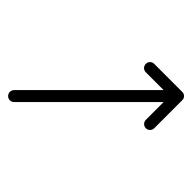 Coping, Escapism, and Fantasy motives | 0.044 (0.037) | .233 | -0.028-0.118 | 0.044 (0.037) | .233 | -0.028-0.117 |

Note. *β* = standardized regression coefficients; S.E. = associated standard error value. GDT = The Gaming Disorder Test (WHO), IGDT-10 = Ten-Item Internet Gaming Disorder Test (APA). ^a^Coded as: 0 = Women, 1 = Men.

*Serial multiple mediation model with covariates*

The results showed that the two serial multiple mediation models, Model_SerialGDTC_ and Model_SerialIGDT-10C_, fit the data well. Model_SerialGDTC_, including the GDT as a measure of gaming disorder symptoms with covariates, showed the following fit indices: χ^2^ = 1897.081, df = 834, p < 0.001; CFI = 0.964; TLI = 0. 0.961; RMSEA = 0.044 [0.041– 0.046, p = 1.000]; and SRMR = 0.052. Model_SerialIGDT-10C_, including the IGDT-10 as a measure of gaming disorder symptoms with covariates showed the following fit statistics: χ^2^ = 2020.733, df = 1054, p < 0.001; CFI = 0.967; TLI = 0.965; RMSEA = 0.037 [0.035– 0.040, p = 1.000]; and SRMR = 0.061. The results from the Model_SerialGDTC_ showed that emotion dysregulation explained 0.72.0% of the variance of self-reported depression symptoms, that self-reported emotion dysregulation and depression symptoms explained 0.45.6% of the variance of CEF motives, and that the variables in the model explained 0.52.5% of gaming disorder symptoms variance. The results from the Model_SerialIGDT-10C_ showed that emotion dysregulation explained 72.0% of the variance of self-reported depression symptoms, that self-reported emotion dysregulation and depression symptoms explained 45.4% of the variance of CEF motives, and that the variables in the model explained 50.8% of gaming disorder symptoms variance.

**Figure B2**

*Visual overview of the serial multiple mediation model, including covariates*


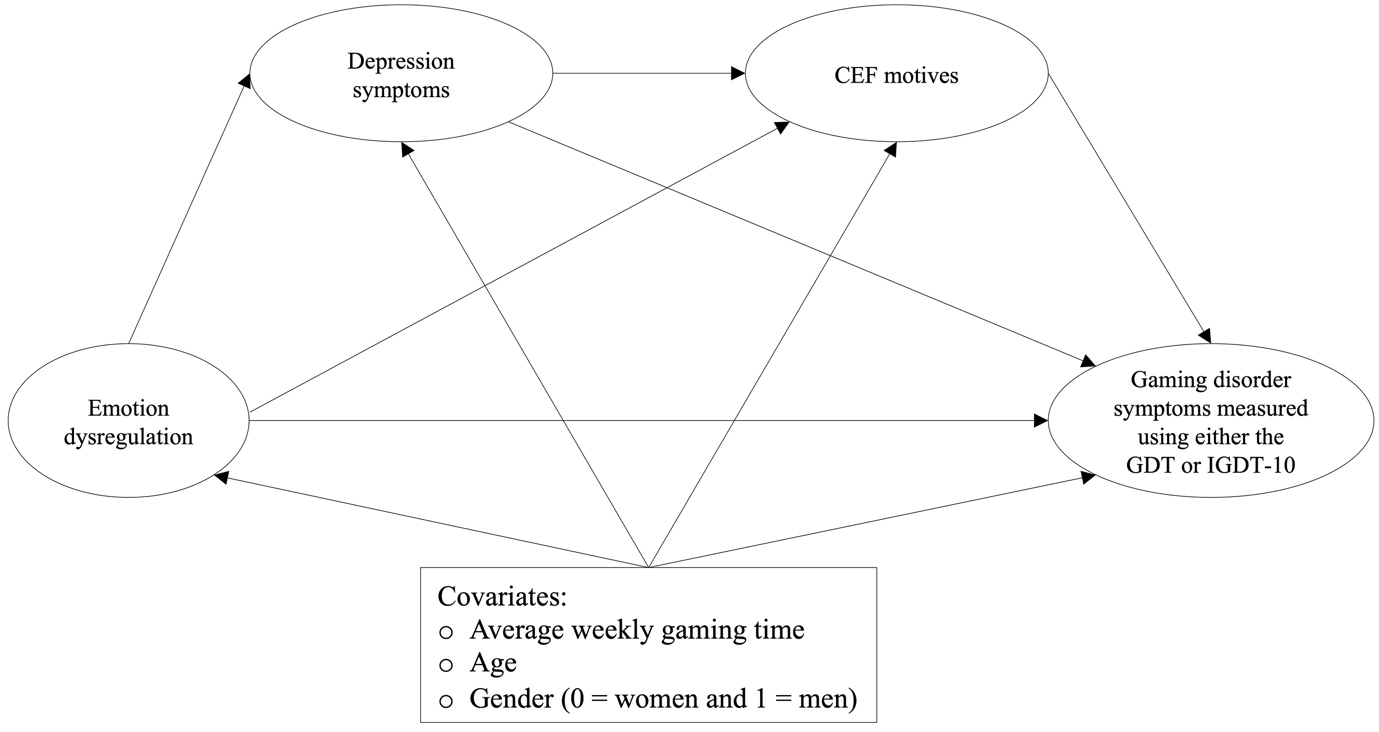


*Note*. GDT = The Gaming Disorder Test (WHO), IGDT-10 = Ten-Item Internet Gaming Disorder Test (APA), CEF motives = Coping, Escapism, and Fantasy motives.

**Table B2**

*Serial multiple mediation model including covariates (5000 bootstrap) (N = 665)*

|  | Model_SerialGDTC_ | | | Model_SerialIGDT-10C_ | | |
| --- | --- | --- | --- | --- | --- | --- |
| **Path coefficients** | Estimate *β* (S.E.) | *p* value | 95% CI | Estimate *β* (S.E.) | *p*  value | 95% CI |
| Emotion dysregulation 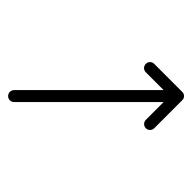 Depression symptoms | 0.831 (0.022) | < .001 | 0.783-0.875 | 0.831 (0.022) | < .001 | 0.782-0.875 |
| Emotion dysregulation 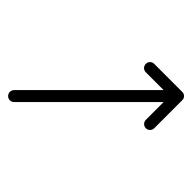 Coping, Escapism, and Fantasy motives | 0.240 (0.080) | .003 | 0.055-0.420 | 0.239 (0.079) | .003 | 0.054-0.418 |
| Emotion dysregulation 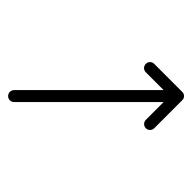 Gaming disorder symptoms | 0.212 (0.087) | .015 | 0.002-0.392 | 0.315 (0.119) | .008 | 0.054-0.561 |
| Depression symptoms 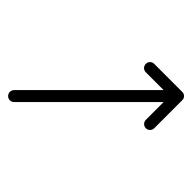 Coping, Escapism, and Fantasy motives | 0.402 (0.079) | < .001 | 0.228-0.579 | 0.402 (0.079) | < .001 | 0.228-0.577 |
| Depression symptoms 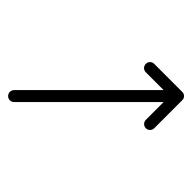 Gaming disorder symptoms | 0.286 (0.088) | .001 | 0.096-0.486 | 0.041 (0.117) | .727 | -0.200-0.285 |
| Coping, Escapism, and Fantasy motives 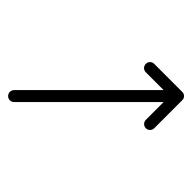 Gaming disorder symptoms | 0.203 (0.051) | < .001 | 0.097-0.318 | 0.352 (0.059) | < .001 | 0.227-0.486 |
| **Indirect Effects** |  |  |  |  |  |  |
| Emotion dysregulation 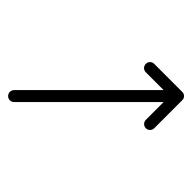 Depression symptoms 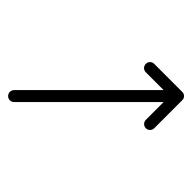 Gaming disorder symptoms | 0.237 (0.074) | .001 | 0.080-0.411 | 0.034 (0.097) | .727 | -0.167-0.240 |
| Emotion dysregulation 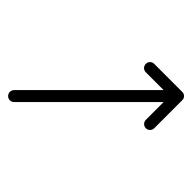 Coping, Escapism, and Fantasy motives 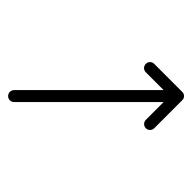 Gaming disorder symptoms | 0.049 (0.021) | .021 | 0.009-0.105 | 0.084 (0.033) | .010 | 0.018-0.168 |
| Emotion dysregulation 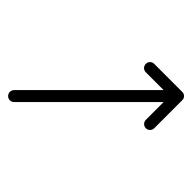 Depression symptoms 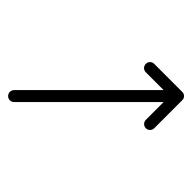 Coping, Escapism, and Fantasy motives 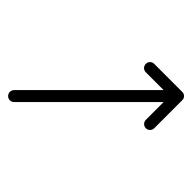 Gaming disorder symptoms | 0.068 (0.021) | .001 | 0.027-0.121 | 0.117 (0.030) | < .001 | 0.060-0.190 |
| **Total effects** |  |  |  |  |  |  |
| Total effects | 0.566 (0.032) | < .001 | 0.488-0.637 | 0.550 (0.044) | < .001 | 0.459-0.644 |
| Total indirect | 0.354 (0.074) | < .001 | 0.201-0.536 | 0.235 (0.101) | .021 | 0.028-0.461 |
| **Covariates** |  |  |  |  |  |  |
| Average weekly gaming time 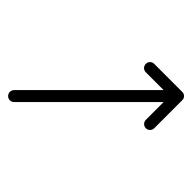 Gaming disorder symptoms | 0.156 (0.035) | < .001 | 0.074-0.238 | 0.157 (0.045) | < .001 | 0.059-0.249 |
| Average weekly gaming time 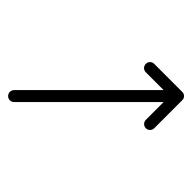 Emotion dysregulation | 0.236 (0.035) | < .001 | 0.157-0.310 | 0.236 (0.035) | < .001 | 0.157-0.310 |
| Average weekly gaming time 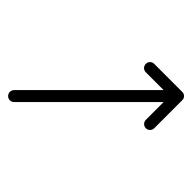 Coping, Escapism, and Fantasy motives | 0.168 (0.031) | < .001 | 0.101-0.236 | 0.168 (0.031) | < .001 | 0.101-0.235 |
| Average weekly gaming time 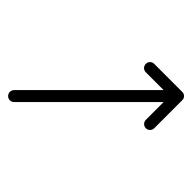 Depression symptoms | 0.086 (0.031) | .005 | 0.023-0.150 | 0.086 (0.031) | .005 | 0.024-0.150 |
| Age 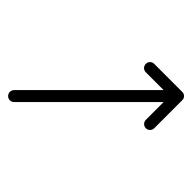 Gaming disorder symptoms | -0.082 (0.036) | .022 | -0.156-(-0.010) | -0.078 (0.051) | .126 | -0.186-0.009 |
| Age 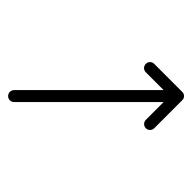 Emotion dysregulation | -0.153 (0.40) | < .001 | -0.235-(-0.076) | -0.153 (0.040) | < .001 | -0.235-(-0.076) |
| Age 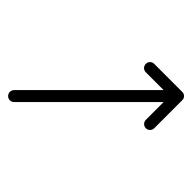 Coping, Escapism, and Fantasy motives | 0.054 (0.036) | .131 | -0.013-0.125 | 0.054 (0.036) | .131 | -0.013-0.125 |
| Age 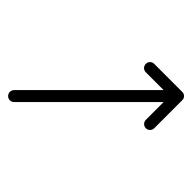 Depression symptoms | 0.017 (0.029) | .551 | -0.040-0.078 | 0.017 (0.029) | .552 | -0.040-0.078 |
| Gender 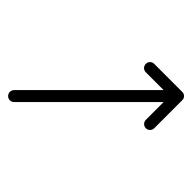 Gaming disorder symptoms | 0.176 (0.033) | < .001 | 0.110-0.241 | 0.086 (0.041) | .035 | 0.009-0.169 |
| Gender 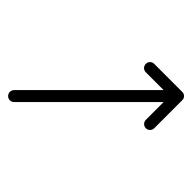 Emotion dysregulation | -0.171 (0.040) | < .001 | -0.247-(-0.089) | -0.171 (0.040) | < .001 | -0.247-(-0.089) |
| Gender 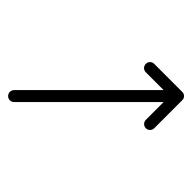 Coping, Escapism, and Fantasy motives | 0.029 (0.035) | .409 | -0.041-0.099 | 0.029 (0.035) | .409 | -0.041-0.099 |
| Gender 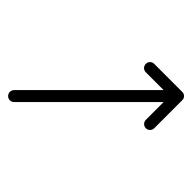 Depression symptoms | 0.035 (0.028) | .210 | -0.021-0.090 | 0.035 (0.028) | .212 | -0.021-0.090 |

*Note*. Standardized coefficients (Estimate β) and the corresponding standard error (S.E.); GDT = The Gaming Disorder Test (WHO); IGDT-10 = Ten-Item Internet Gaming Disorder Test (APA). ^a^Coded as: 0 = Women, 1 = Men.

**Sensitivity analyses 2: mediation models including the IGDT-10_without item 8_**

*Sensitivity analyses of the full measurement model*

The full measurement model when gaming disorder symptoms were measured with the **IGDT-10** without item 8 showed excellent model fit to the data (*χ^2^* = 1900.745, df = 1008, p < .0001; CFI = 0.972; TLI = 0.970; RMSEA = 0.036 [0.034 - 0.039, *p* = 1.000], and SRMR = 0.055).

**Table C1**

*Standardized correlations from the sensitivity analyses of the full measurement model (n = 678)*

|  | 1 | 2 | 3 | 4 | 5 | 6 |
| --- | --- | --- | --- | --- | --- | --- |
| 1. IGDT-10 | - |  |  |  |  |  |
| 2. Emotion dysregulation | 0.547^***^ | - |  |  |  |  |
| 3. Depression symptoms | 0.535^***^ | 0.837^***^ | - |  |  |  |
| 4. Coping, Escapism, and Fantasy motives | 0.516^***^ | 0.611^***^ | 0.647^***^ | - |  |  |
| 5. Average weekly gaming time | 0.344^***^ | 0.224^***^ | 0.278^***^ | 0.332^***^ | - |  |
| 6. Age | -0.200^**^ | -0.171^***^ | -0.143^**^ | -0.066 | -0.092^***^ | - |
| 7. Gender^a^ | 0.120 | -0.187^**^ | -0.098 | -0.027 | 0.143^**^ | -0.052 |

*Notes.* ^*^*p* < .05; ^**^ *p* < .01; ^***^ *p* < .001. GDT = The Gaming Disorder Test (WHO), IGDT-10 = Ten-Item Internet Gaming Disorder Test (APA). ^a^Coded as: 0 = Women, 1 = Men.

*Sensitivity simple mediation model without covariates*

The simple mediation model without covariates including **IGDT-10**_without item 8_ provided a good fit to the data (*χ^2^* = 1070.377, df = 583, p < .0001; CFI = 0.982; TLI = 0.980; RMSEA = 0.035 [0.032 - 0.038, *p* = 1.000], and SRMR = 0.053).

**Table C2**

*Sensitivity analyses of mediation model without covariates (5000 bootstrap) (n = 678) (See Figure B1).*

|  | **IGDT-10**_without item 8_ | | |
| --- | --- | --- | --- |
| **Path coefficients including direct effects** | Estimate *β* (S.E.) | *p* value | 95% CI |
| Emotion dysregulation 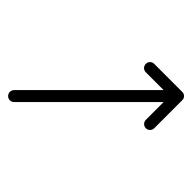 Coping, Escapism, and Fantasy motives | 0.614 (0.032) | < .001 | 0.543-0.682 |
| Emotion dysregulation 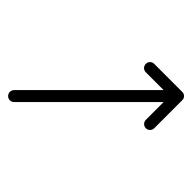 Gaming disorder symptoms | 0.367 (0.070) | < .001 | 0.222-0.513 |
| Coping, Escapism, and Fantasy motives 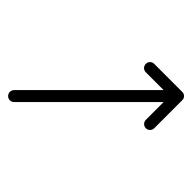 Gaming disorder symptoms | 0.293 (0.068) | < .001 | 0.149-0.439 |
| **Indirect Effects** |  |  |  |
| Emotion dysregulation 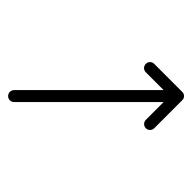 Coping, Escapism, and Fantasy motives 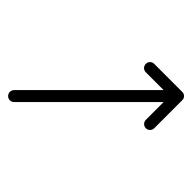 Gaming disorder symptoms | 0.180 (0.044) | < .001 | 0.089-0.280 |
| **Total effects** |  |  |  |
| Emotion dysregulation 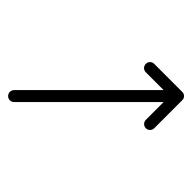 Gaming disorder symptoms | 0.547 (0.045) | < .001 | 0.456-0.639 |

Note. *β* = standardized regression coefficients; S.E. = associated standard error value. IGDT-10 = Ten-Item Internet Gaming Disorder Test.

*Sensitivity mediation model with covariates*

The mediation model with covariates including **IGDT-10** without item 8 provided a good fit to the data (χ^2^ = 1198.795, df = 682, p < .0001; CFI = 0.979; TLI = 0.977; RMSEA = 0.034 [0.031 - 0.037, *p* = 1.000], and SRMR = 0.055).

**Table C3**

*Sensitivity analyses of mediation model with covariates (5000 bootstrap) (n = 665)*

|  | **IGDT-10**_without item 8_ | | |
| --- | --- | --- | --- |
| **Path coefficients** | Estimate *β* (S.E.) | *p*  value | 95% CI |
| Emotion dysregulation 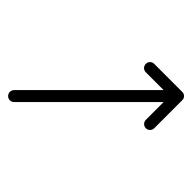 Coping, Escapism, and Fantasy motives | 0.576 (0.035) | < .001 | 0.497-0.651 |
| Emotion dysregulation 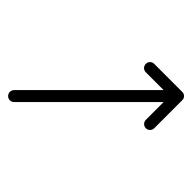 Gaming disorder symptoms | 0.392 (0.071) | < .001 | 0.234-0.548 |
| Coping, Escapism, and Fantasy motives 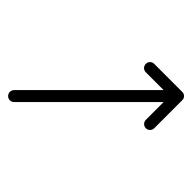 Gaming disorder symptoms | 0.207 (0.071) | .003 | 0.055-0.371 |
| **Indirect Effects** |  |  |  |
| Emotion dysregulation 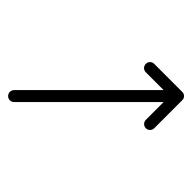 Coping, Escapism, and Fantasy motives 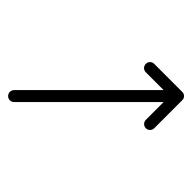 Gaming disorder symptoms | 0.119 (0.042) | .005 | 0.032-0.219 |
| **Total effects** |  |  |  |
| Emotion dysregulation 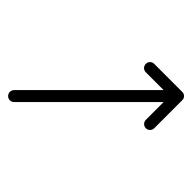 Gaming disorder symptoms | 0.511 (0.048) | < .001 | 0.408-0.614 |
| **Covariates** |  |  |  |
| Average weekly gaming time 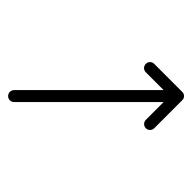 Gaming disorder symptoms | 0.186 (0.048) | < .001 | 0.080-0.284 |
| Average weekly gaming time 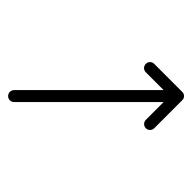 Emotion dysregulation | 0.237 (0.035) | < .001 | 0.158-0.310 |
| Average weekly gaming time 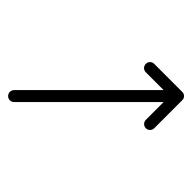 Coping, Escapism, and Fantasy motives | 0.203 (0.032) | < .001 | 0.132-0.275 |
| Age 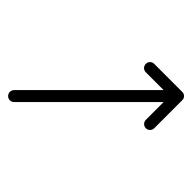 Gaming disorder symptoms | -0.101 (0.059) | .084 | -0.222-(-0.002) |
| Age 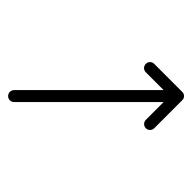 Emotion dysregulation | -0.153 (0.040) | < .001 | -0.234-(-0.076) |
| Age 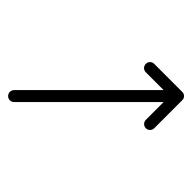 Coping, Escapism, and Fantasy motives | 0.062 (0.037) | .099 | -0.011-0.136 |
| Gender 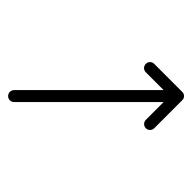 Gaming disorder symptoms | 0.115 (0.046) | .014 | 0.029-0.211 |
| Gender 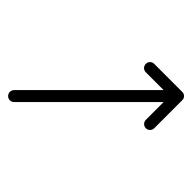 Emotion dysregulation | -0.172 (0.040) | < .001 | -0.248-(-0.089) |
| Gender 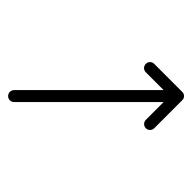 Coping, Escapism, and Fantasy motives | 0.044 (0.037) | .236 | -0.028-0.117 |

Note. *β* = standardized regression coefficients; S.E. = associated standard error value. IGDT-10 = Ten-Item Internet Gaming Disorder Test. ^a^Coded as: 0 = Women, 1 = Men.

*Sensitivity serial multiple mediation model without covariates*

The serial mediation model, including **IGDT-10** without item 8 and without covariates, provided a good fit to the data (*χ^2^* = 1739.823, df = 888, p < .0001; CFI = 0.973; TLI = 0.971; RMSEA = 0.038 [0.035 - 0.040, *p* = 1.000], and SRMR = 0.055).

**Table C4**

*Sensitivity analyses of serial multiple mediation model without covariates (5000 bootstrap) (n = 678) (See Figure B2)*

|  | **IGDT-10**_without item 8_ | | |
| --- | --- | --- | --- |
| **Path coefficients including direct effects** | Estimate *β* (S.E.) | *p*  value | 95% CI |
| Emotion dysregulation 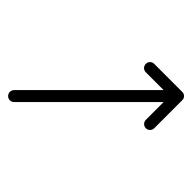 Depression symptoms | 0.837 (0.018) | < .001 | 0.797-0.874 |
| Emotion dysregulation 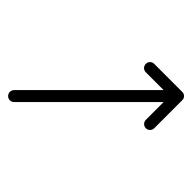 Coping, Escapism, and Fantasy motives | 0.231 (0.078) | .003 | 0.057-0.399 |
| Emotion dysregulation 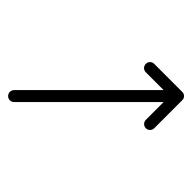 Gaming disorder symptoms | 0.272 (0.119) | .023 | 0.019-0.520 |
| Depression symptoms 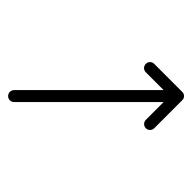 Coping, Escapism, and Fantasy motives | 0.454 (0.077) | < .001 | 0.287-0.620 |
| Depression symptoms 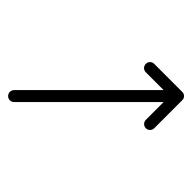 Gaming disorder symptoms | 0.139 (0.123) | .257 | -0.107-0.400 |
| Coping, Escapism, and Fantasy motives 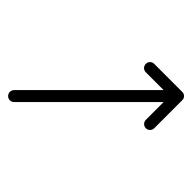 Gaming disorder symptoms | 0.260 (0.069) | < .001 | 0.107-0.411 |
| **Indirect Effects** |  |  |  |
| Emotion dysregulation 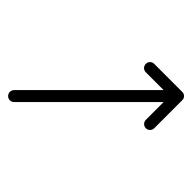 Depression symptoms 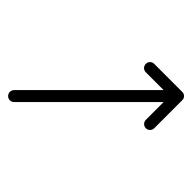 Gaming disorder symptoms | 0.117 (0.103) | .258 | -0.091-0.339 |
| Emotion dysregulation 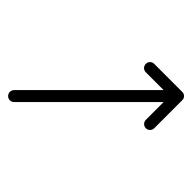 Coping, Escapism, and Fantasy motives 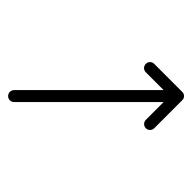 Gaming disorder symptoms | 0.060 (0.027) | .026 | 0.010-0.132 |
| Emotion dysregulation 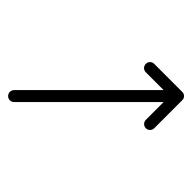 Depression symptoms 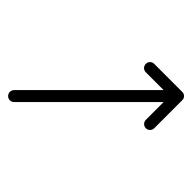 Coping, Escapism, and Fantasy motives 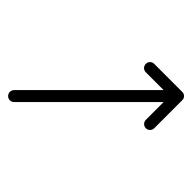 Gaming disorder symptoms | 0.099 (0.031) | .001 | 0.039-0.173 |
| **Total effects** |  |  |  |
| Total effects | 0.547 (0.045) | < .001 | 0.457-0.639 |
| Total indirect | 0.276 (0.101) | .006 | 0.070-0.490 |

Note. *β* = standardized regression coefficients; S.E. = associated standard error value. IGDT-10 = Ten-Item Internet Gaming Disorder Test.

*Sensitivity serial multiple mediation model with covariates*

The serial mediation model, including the **IGDT-10** without item 8 and with covariates, provided a good fit to the data (*χ^2^* = 1862.973, df = 1008, p < .0001; CFI = 0.971; TLI = 0.969; RMSEA = 0.036 [0.033 - 0.038, *p* = 1.000], and SRMR = 0.057).

**Table C5**

*Sensitivity analyses of the serial multiple mediation model with covariates (5000 bootstrap) (n = 665)*

|  | **IGDT-10**_without item 8_ | | |
| --- | --- | --- | --- |
| **Path coefficients including direct effects** | Estimate *β* (S.E.) | *p*  value | 95% CI |
| Emotion dysregulation 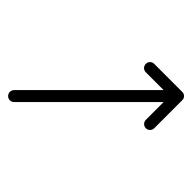 Depression symptoms | 0.831 (0.022) | 0.000 | 0.782-0.875 |
| Emotion dysregulation 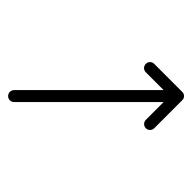 Coping, Escapism, and Fantasy motives | 0.240 (0.080) | 0.003 | 0.055-0.420 |
| Emotion dysregulation 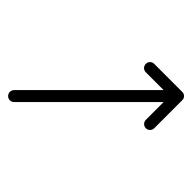 Gaming disorder symptoms | 0.339 (0.129) | 0.009 | 0.051-0.606 |
| Depression symptoms 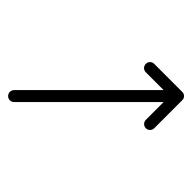 Coping, Escapism, and Fantasy motives | 0.402 (0.079) | 0.000 | 0.228-0.579 |
| Depression symptoms 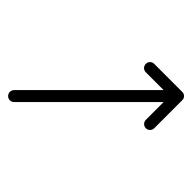 Gaming disorder symptoms | 0.077 (0.128) | 0.547 | -0.187-0.363 |
| Coping, Escapism, and Fantasy motives 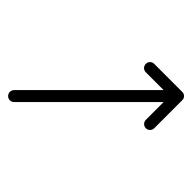 Gaming disorder symptoms | 0.189 (0.069) | 0.006 | 0.039-0.351 |
| **Indirect Effects** |  |  |  |
| Emotion dysregulation 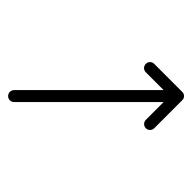 Depression symptoms 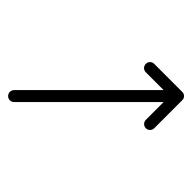 Gaming disorder symptoms | 0.064 (0.106) | 0.547 | -0.158-0.306 |
| Emotion dysregulation 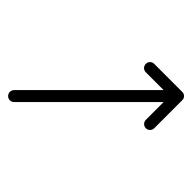 Coping, Escapism, and Fantasy motives 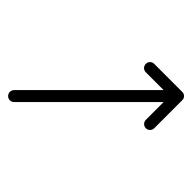 Gaming disorder symptoms | 0.045 (0.024) | 0.056 | 0.003-0.114 |
| Emotion dysregulation 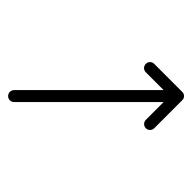 Depression symptoms 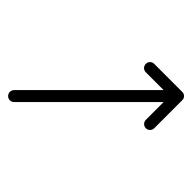 Coping, Escapism, and Fantasy motives 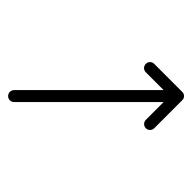 Gaming disorder symptoms | 0.063 (0.025) | 0.013 | 0.013-0.125 |
| **Total effects** |  |  |  |
| Total effects | 0.511 (0.048) | 0.000 | 0.408-0.615 |
| Total indirect | 0.173 (0.108) | 0.112 | -0.054-0.419 |
| **Covariates** |  |  |  |
| Average weekly gaming time 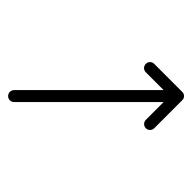 Gaming disorder symptoms | 0.183 (0.050) | 0.000 | 0.075-0.282 |
| Average weekly gaming time 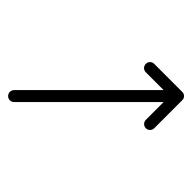 Emotion dysregulation | 0.236 (0.035) | 0.000 | 0.157-0.310 |
| Average weekly gaming time 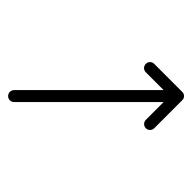 Coping, Escapism, and Fantasy motives | 0.168 (0.031) | 0.000 | 0.101-0.236 |
| Average weekly gaming time 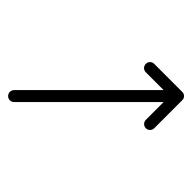 Depression symptoms | 0.086 (0.031) | 0.005 | 0.024-0.150 |
| Age 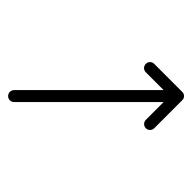 Gaming disorder symptoms | -0.101 (0.059) | 0.086 | -0.222-0.000 |
| Age 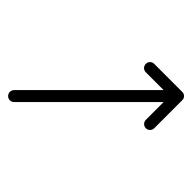 Emotion dysregulation | -0.153 (0.040) | 0.000 | -0.235-(-0.076) |
| Age 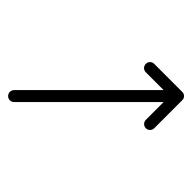 Coping, Escapism, and Fantasy motives | 0.054 (0.036) | 0.131 | -0.013-0.125 |
| Age Depression symptoms | 0.017 (0.029) | 0.553 | -0.040-0.078 |
| Gender Gaming disorder symptoms | 0.113 (0.046) | 0.015 | 0.026-0.208 |
| Gender Emotion dysregulation | -0.171 (0.040) | 0.000 | -0.247-(-0.089) |
| Gender Coping, Escapism, and Fantasy motives | 0.029 (0.035) | 0.411 | -0.041-0.099 |
| Gender Depression symptoms | 0.035 (0.028) | 0.212 | -0.021-0.090 |

*Note*. Standardized coefficients (Estimate *β*) and the corresponding standard error (S.E.). IGDT-10 = Ten-Item Internet Gaming Disorder Test. ^a^Coded as: 0 = Women, 1 = Men.
